# TACI expression and plasma cell differentiation are impaired in the absence of functional IκBNS

**DOI:** 10.1111/imcb.12228

**Published:** 2019-01-30

**Authors:** Sharesta Khoenkhoen, Elina Erikson, Monika Ádori, Julian M Stark, Jean L Scholz, Michael P Cancro, Gabriel K Pedersen, Gunilla B Karlsson Hedestam

**Affiliations:** ^1^ Department of Microbiology, Tumor and Cell Biology Karolinska Institutet Stockholm Sweden; ^2^ Perelman School of Medicine University of Pennsylvania Philadelphia PA USA

**Keywords:** *APRIL*, *IκBNS*, *nfkbid*, *NF‐κB*, plasma cell differentiation, *TACI*

## Abstract

Impaired classical NF‐κB pathway signaling causes reduced antibody responses to T‐independent (TI) antigens. We investigated the potential reasons for defective TI responses in mice lacking the atypical inhibitory kappa B (IκB) protein of the NF‐κB pathway, IκBNS. Analyses of the plasma cell compartment *in vitro* and *in vivo* after challenge with lipopolysaccharide (LPS) showed significant decreases in the frequencies of plasma cells in the absence of IκBNS. *In vitro* activation of B cells via the B cell receptor or via Toll‐like receptor 4 revealed that early activation events were unaffected in IκBNS‐deficient B cells, while proliferation was reduced compared to in similarly stimulated wildtype (wt) B cells. IκBNS‐deficient B cells also displayed impaired upregulation of the transmembrane activator and calcium modulator cyclophilin ligand interactor (TACI), which is essential for TI responses, and decreased sensitivity to TACI ligands upon stimulation. Furthermore, IκBNS‐deficient B cells, in contrast to wt B cells, displayed altered expression of IRF4, Blimp‐1 and Pax5 upon LPS‐induced differentiation, indicating impaired transcriptional regulation of plasma cell generation.

## Graphical abstract

This study identified alterations in B cell activation and plasma cell differentiation as underlying reasons for the impaired humoral responses against T‐independent (TI) antigens associated with IκBNS deficiency. In the absence of IκBNS, B cells exhibited reduced proliferative capacity, a loss of TACI‐mediated antibody production, and altered expression of the transcription factors Pax5, IRF4 and Blimp‐1, and fail to generate plasma cells. These findings indicate that TACI function and plasma cell differentiation are regulated by IκBNS, and may help explain mechanisms for impaired TI responses associated with defects in components of the NF‐κB signaling pathway.

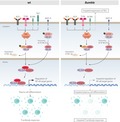

## Introduction

The activation and differentiation of B lymphocytes into plasma cells (PC) and their subsequent production of antibodies is pivotal for establishing efficient humoral immune responses to infections. After ligation of their B cell receptors (BCR), B cells are able to differentiate into antibody‐secreting PC or memory B cells either with or without T‐cell help, resulting in T‐dependent (TD) or T‐independent (TI) antibody responses, respectively. The TI antigens are classified according to whether they are capable of stimulating antibody production in mice with reduced BCR signaling due to Bruton's tyrosine kinase (*xid*/*btk)* deficiency (TI‐1) or not (TI‐2).^1^ Thus, intact BCR signaling machinery is required for responses to TI‐2 antigens. The TI‐1 antigens stimulate B cells by binding to both BCR and pathogen recognition receptors such as Toll‐like receptors (TLR), while TI‐2 antigens display repetitive determinants, usually composed of polysaccharides, which activate B cells via BCR ligation.^2^ The TNF superfamily ligands, B cell activating factor (BAFF/BLyS) and a proliferation inducing ligand (APRIL), have been implicated in the response to TI antigens. While BAFF and APRIL also signal through the BAFF receptor (BAFFR) and/or BCMA, it is their ligation to the transmembrane activator and calcium modulator cyclophilin ligand interactor (TACI) that is considered essential for TI antibody responses.^3^, ^4^, ^5^

The TI antigens are found primarily on the surface of encapsulated bacteria such as *Streptococcus pneumoniae* and *Haemophilus influenzae*. These bacteria are among the most prevalent microorganisms causing recurrent respiratory tract infections in patients with primary antibody deficiencies (PAD).^6^ Approximately 8–10% of patients with the most common PAD, combined variable immunodeficiency (CVID), have homo‐ or heterozygous mutations in TACI. However, there is no clear clinical phenotype associated with specific TACI mutations, suggesting that additional environmental or genetic factors contribute to TACI deficiency‐associated CVID.^7^, ^8^ BCR and TLR stimulation also induce NF‐κB activation and dysfunctional NF‐κB signaling is associated with various defects in B cell function,^9^ which potentially manifests as CVID.^10^, ^11^, ^12^, ^13^

The NF‐κB transcription factors, p50 (NF‐κB1), p52 (NF‐κB2), p65 (RelA), c‐Rel and RelB, regulate transcription by binding to promoters of target genes. In classical NF‐κB signaling, the NF‐κB transcription factors are rendered transcriptionally inactive through sequestering in the cytoplasm by inhibitors of κB (IκB), such as IκB‐α, IκB‐β, IκB‐ε, and the p50 precursor p105.^14^ In addition to the cytoplasmic IκB proteins, the atypical nuclear IκB proteins BCL‐3, IκBζ, IκBNS and IκBη were identified based on their ankyrin repeat structure through which they are able to bind NF‐κB proteins and regulate their activity.^15^ For example, IκBNS was found to bind nuclear p50, p52, p65, RelB and c‐Rel.^16^, ^17^ Rather than being constitutively expressed and regulated through proteasomal degradation similar to classical IκB proteins, expression of IκBNS is induced by BCR ligation or TLR stimulation.^18^, ^19^

Recent studies have revealed distinct functions of atypical IκB proteins in lymphopoiesis and immunological responses (reviewed in 20). For instance, BCL‐3 deficiency leads to increased marginal zone B (MZB) cell numbers and fewer follicular B (FOB) cells,^21^ whereas the phenotype of IκBNS‐deficient mice resembles other strains with classical NF‐κB pathway deficiency. Similar to p50^−/−^ and c‐Rel^−/−^ mice, the MZB and B‐1 cell numbers are reduced and serum IgM and IgG3 levels are decreased in IκBNS^−/−^ mice.^19^ The IκBNS‐deficient *bumble* (*bmb*) strain harbors a mutation in the donor splice site of intron 4 within the *nfkbid* gene, which introduces a premature stop codon in the transcript and encodes for a severely truncated IκBNS protein that is not expected to retain any function.^18^ Similar to IκBNS knock‐out mice, the *bumble* mice completely lack the B‐1a cell population,^18^, ^22^ while in p50^−/−^ mice this population is only reduced.^23^ Development of the B‐1a population via the neonatal transitional B‐1a (TrB‐1a) cell stage and MZB population via the transitional‐2 marginal zone precursor stage depends on IκBNS.^22^, ^24^ Furthermore, the *bumble* mice are unable to respond to TI antigens while heterozygous *bumble* mice are haploinsufficient in terms of TI antibody responses despite intact B cell development.^25^ These results indicated that IκBNS is required for normal antibody responses to TI antigens, in addition to its role in B cell development.

IκBNS is also required for normal function in other immune cells. In T cells, IκBNS mediates TCR‐induced cell death during negative selection in the thymus,^16^ governs the development of regulatory T cells through the induction of Foxp3^20^ and is essential for cytokine production in T_H_17 cells.^15^ In the myeloid lineage, IκBNS dampens the proinflammatory response through suppression of IL‐6 and IL‐12p40 production in macrophages and regulating IL‐10 production by dendritic cells upon lipopolysaccharide (LPS) stimulation.^26^, ^27^, ^28^

In this study, we investigated potential reasons for the lack of TI responses in the absence of IκBNS using the *bumble* mouse strain.^18^ We found that *bumble* B cells displayed impaired expression of TACI, both at steady‐state and in response to stimulation, as well as reduced responses to the TACI ligands APRIL and BAFF. A comparison of LPS‐stimulated B cell cultures from *bumble* and wildtype (wt) mice revealed altered expression of the transcription factors Pax5, IRF4 and Blimp‐1, all of which coordinate PC differentiation. These findings demonstrate that IκBNS deficiency is associated with both impaired TACI expression and defective transcriptional regulation of PC differentiation.

## Results

### PC generation in response to the T‐independent antigen LPS requires functional IκBNS

**Figure 1:**
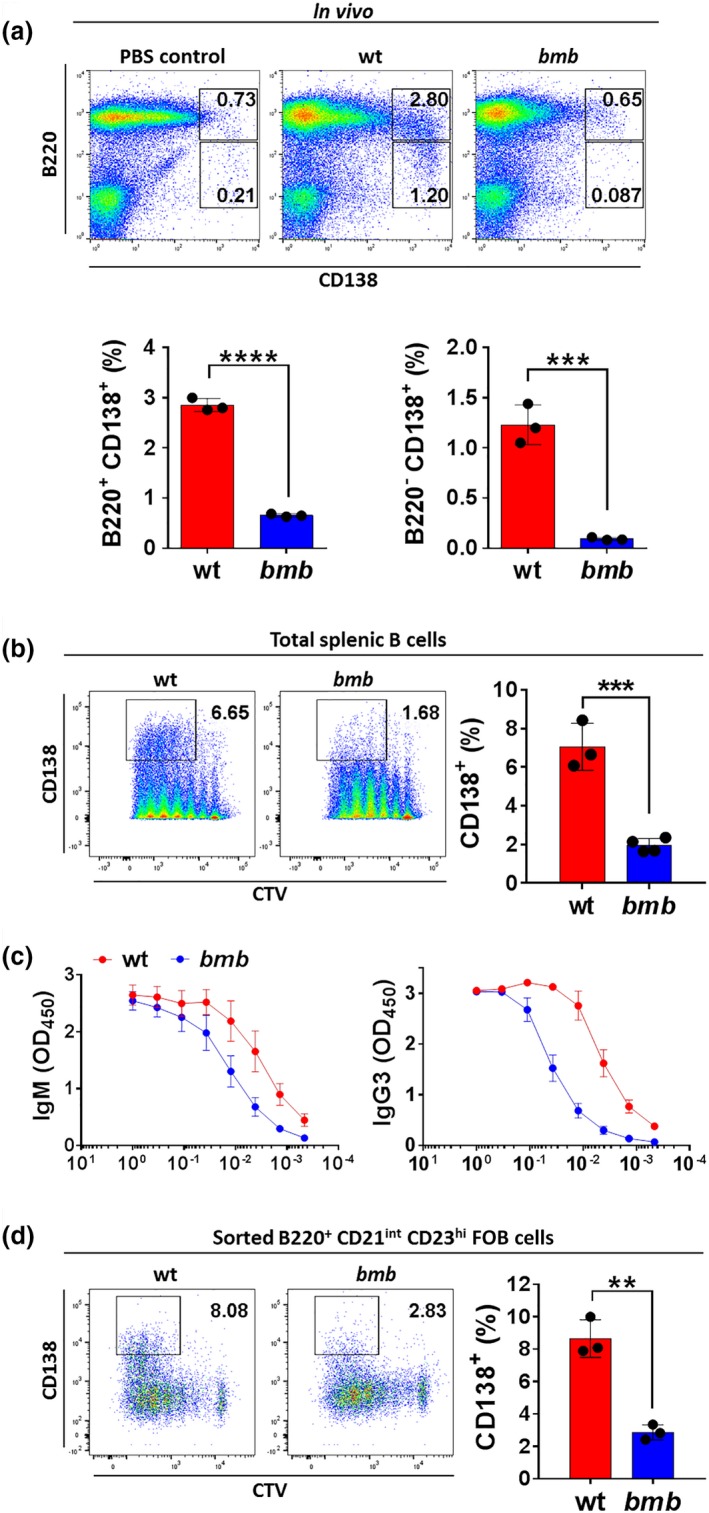
Plasma cell generation in response to the T‐independent antigen LPS requires functional IκBNS. LPS‐induced PC generation in wt mice and IκBNS‐deficient *bumble* (*bmb*) mice. **(a)** Wt and *bumble* mice were injected with 5 μg *S. minnesota* LPS intravenously and plasmablast and plasma cell frequencies were determined at day 3. Representative plots show the B220^+^ CD138^+^ plasmablast and the B220^−^ CD138^+^ plasma cell populations (upper panel). Frequencies of plasmablasts and plasma cells in LPS‐immunized wt and *bumble* mice (lower panel). Graph bars and error bars indicate mean ± s.d. Differences between groups were determined using an unpaired Student's *t*‐test with *** and **** indicating *P* ≤ 0.001 and *P* ≤ 0.0001, respectively. Data are representative of two independent experiments with 3 wt and 3 *bumble* mice. **(b)** Isolated splenic B cells from wt and *bumble* mice were CTV‐labeled, stimulated with 10 μg mL^−1^ LPS, and stained for CD138 expression at 84 h. Representative plots show the gate for identifying cells undergoing plasma cell differentiation (left panel). Frequencies of CD138^+^ cells in wt and *bumble* B cell cultures (right panel). Graph bars and error bars indicate mean ± s.d. Differences between groups were determined using an unpaired Student's *t*‐test with *** indicating *P* ≤ 0.001. Data are representative of three independent experiments with 3–5 mice in each group. **(c)** Isolated splenic B cells from wt and *bumble* mice were stimulated with 10 μg mL^−1^ LPS for 6 days. Levels of IgM and IgG3 were determined by ELISA from the supernatant of wt and *bumble* B cell cultures. Each line and error bar indicate mean ± s.d. Data are representative of five independent experiments with 3–5 mice in each group. **(d)** Sorted B220^+^ CD21^int^ CD23^hi^ FOB cells from wt and *bumble* mice were CTV‐labeled and stimulated with 10 μg mL^−1^ LPS, and stained for CD138 expression at 84 h (left panel). Graph bars and error bars indicate mean ± s.d. Statistical differences were determined using an unpaired Student's *t*‐test with ** indicating *P* ≤ 0.01. Data are representative of two independent experiments with 3 wt and 3 *bumble* mice.

We previously reported a requirement for IκBNS for intact antibody responses to TI antigens.^18^, ^22^, ^25^ TI antigens stimulate rapid extrafollicular plasmablast and PC responses.^29^ *bumble* mice displayed impaired antibody responses to immunization with the TI‐1 antigen 2,4,6‐trinitrophenyl (TNP)‐LPS and the TI‐2 antigens NP (4‐hydroxy‐3‐nitrophenylacetic)‐Ficoll and Pneumococcal polysaccharides (Pneumovax).^22^ To investigate the role of IκBNS for antibody induction, we assessed PC generation in response to the TLR4 ligand LPS, which provides a TI‐1 antigen stimulus. We first examined the splenic plasmablast and PC compartments *in vivo* after injection with 5 μg LPS i.v. We found that the frequencies of both B220^+^ CD138^+^ plasmablasts and B220^−^ CD138^+^ PC were reduced significantly in *bumble* mice compared to in wt mice (Figure 1a). We also examined PC generation *in vitro*. B cells committing to PC fate upregulate CD138 after approximately four division cycles.^30^ Therefore, we labeled isolated splenic B cells with the cell trace violet (CTV) dye, cultured them with LPS for 84 h (3.5 days), and then stained the cells for CD138 expression. LPS stimulation resulted in efficient generation of CD138^+^ cells in wt, but not in *bumble* B cell cultures (Figure 1b). The reduction in CD138^+^ cell frequencies in *bumble* B cell cultures was accompanied by reduced secretion of IgM and IgG3 into the culture supernatant (Figure 1c). In addition, we stimulated sorted FOB cells (purity approximately 99%, Supplementary figure 1) to exclude the possibility that the reduction in CD138^+^ cells was influenced by the decreased MZB compartment in *bumble* mice.^18^, ^24^ Similar to the cultures from total splenic B cells, the frequencies of CD138^+^ cells from sorted FOB cells were reduced in *bumble* cultures compared to in wt cultures (Figure 1d). Thus, IκBNS is required for intact generation of plasmablast and PC responses to the TI‐1 antigen LPS.

### Early activation events are modestly altered in IκBNS‐deficient B cells

**Figure 2:**
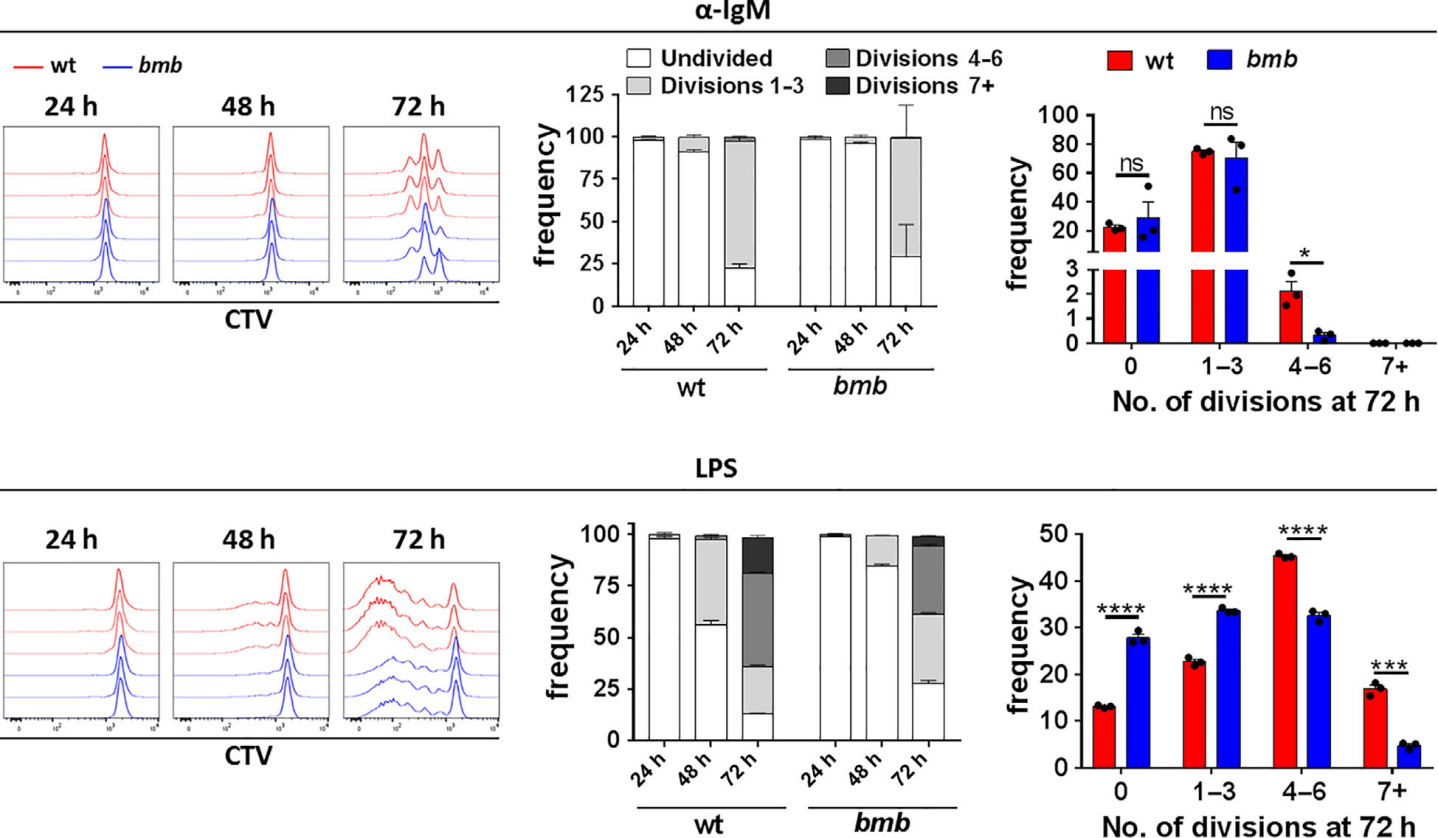
*bumble* B cells display reduced proliferation in response to BCR or TLR4 stimulation. Isolated B cells from spleen of wt and *bumble* mice were labeled with CTV, and stimulated with 10 μg mL^−1^ anti‐IgM F(ab’)_2_ fragments (upper panel) or 10 μg mL^−1^ LPS (lower panel) for 24, 48 and 72 h. Proliferation in response to anti‐IgM or LPS stimulation is indicated by CTV dilution. Representative histograms show proliferation profiles from 3 wt and 3 *bumble* samples (left panels). Frequencies of wt and *bumble* B cells at various phases of cellular division are summarized for 24, 48 and 72 h (middle panels). Bars and error bars indicate mean ± s.d. Frequencies of wt and *bumble* B cells at 72 h (right panels). Graph bars and error bars indicate mean ± s.d. Data are representative of four independent experiments with 3 wt and 3 *bumble* mice. Differences between groups was determined using an unpaired Student's *t*‐test with ns, *, *** and **** indicating *P* > 0.05, *P* ≤ 0.05, *P* ≤ 0.001 and *P* ≤ 0.0001, respectively.

To investigate further the role of IκBNS in TI responses, we assessed whether initial signaling events downstream of BCR ligation were intact in *bumble* mice. To induce BCR signaling, we used anti‐IgM F(ab’)_2_ fragments for BCR ligation to model TI‐2 antigen stimulation. Activated B cells increase in size allowing identification of blasted cells by the forward and side scatter profiles. After activation via BCR or TLR4 ligation, the frequencies of blasted B cells in wt and *bumble* cultures were comparable at 24 h (Supplementary figure 2a). We also observed similar surface expression of the co‐stimulatory protein CD86 on *bumble* and wt B cells (Supplementary figure 2b). As the NF‐κB transcription factors p50/p65 are bound and sequestered by IκB proteins, activation of the classical pathway depends on IκBα phosphorylation and degradation upon BCR signaling. Mice with impaired BCR signaling, for example, because of mutations in *btk,* 31 or genes encoding classical NF‐κB pathway proteins such as NEMO or IKKβ,^32^, ^33^, ^34^ fail to induce IκBα phosphorylation. To assess whether induction of BCR signaling was intact, we measured IκBα degradation in wt and *bumble* B cells. IκBα was degraded normally after 90 min of stimulation with anti‐IgM, PMA and ionomycin or after treatment with the protein phosphatase inhibitor, calyculin A (Supplementary figure 2c). Since differentiation of activated B cells into PC requires several cellular divisions,^30^, ^35^ we next examined proliferation in wt and *bumble* B cells in response to anti‐IgM or LPS. In response to anti‐IgM, only a few cells reached division cycles 4–6 in both wt and *bumble* B cell cultures at 72 h. However, we observed clear decreases in the frequencies of B cells reaching division cycle 4 or beyond in *bumble* compared to in wt B cell cultures at 72 h in response to LPS (Figure**** 2 and Supplementary figure 3). Thus, while the initial activation of B cells was unaffected in terms of blasting, CD86 upregulation, and IκBα degradation, subsequent proliferation events were impaired in *bumble* B cells.

### IκBNS is essential for intact TACI upregulation in response to BCR or TLR stimulation

**Figure 3:**
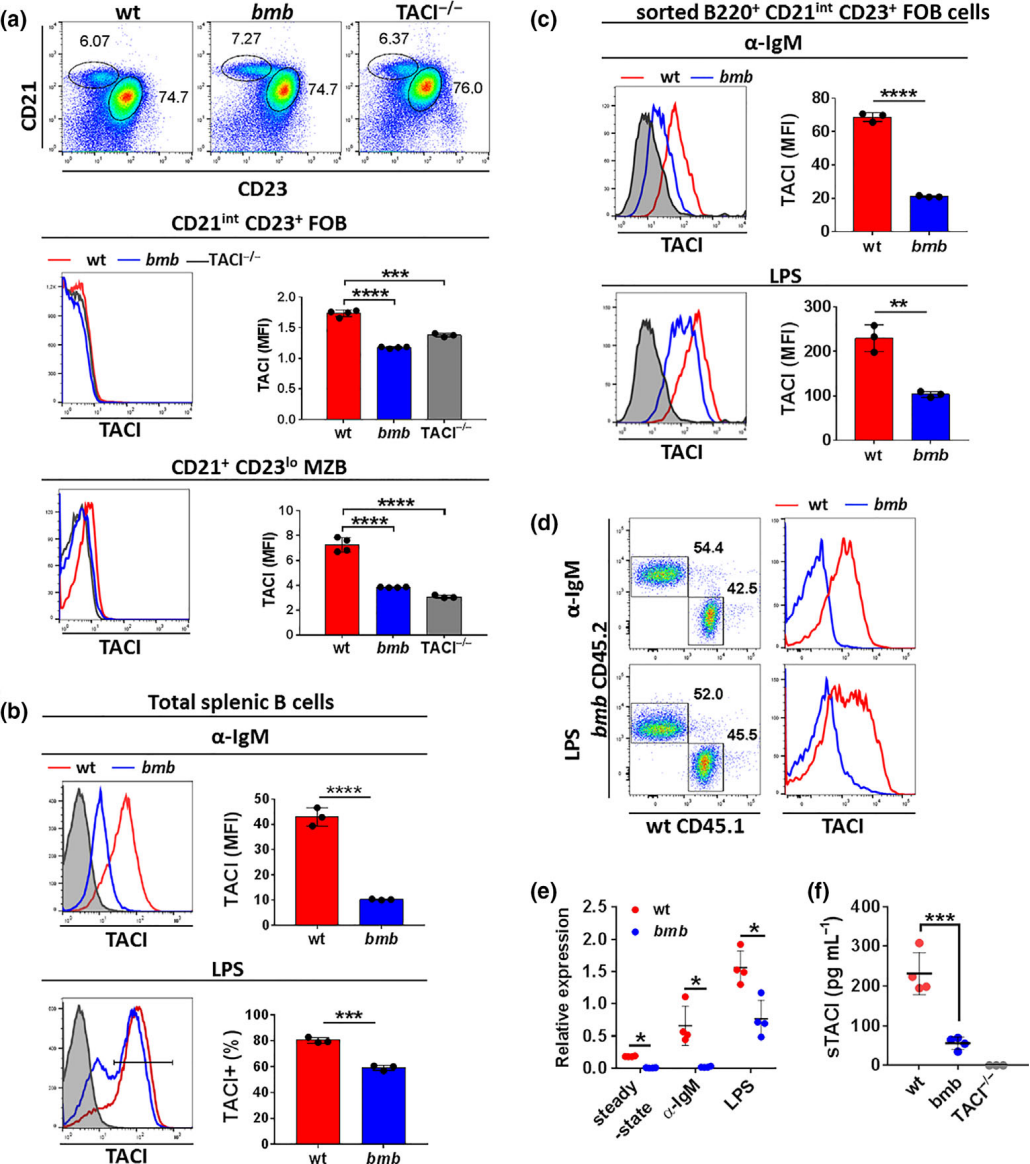
TACI upregulation upon BCR or TLR4 stimulation is impaired in IκBNS‐deficient B cells. **(a)** Follicular B (FOB, B220^+^ CD23^+^ CD21^int^) and marginal zone B (MZB, B220^+^ CD23^−^ CD21^hi^) cells from 6 to 7 months old wt, *bumble* and TACI ^−/−^ mice were stained for TACI by flow cytometry. Representative plots showing the FOB and MZB compartment (upper panel). Numbers adjacent to the gates indicate frequencies. TACI expression on FOB (middle panel), and on MZB cells (lower panel). Data are representative of two independent experiments with 3 or 4 mice in each group. Graph bars and error bars indicate mean ± s.d. Statistical significance was calculated using an unpaired Student's *t*‐test with *** and **** indicating *P* ≤ 0.001 and *P* ≤ 0.0001, respectively. **(b)** Isolated B cells from spleen of wt or *bumble* mice were stimulated with 10 μg mL^−1^ anti‐IgM F(ab’)_2_ fragments (upper panel) or 10 μg mL^−1^ LPS (lower panel) for 48 h. Graph bars and error bars indicate mean ± s.d. Data are representative of four independent experiments with three mice in each group. Statistical significance was calculated using an unpaired Student's *t*‐test with *** and **** indicating *P* ≤ 0.001 and *P* ≤ 0.0001, respectively. **(c)** Sorted FOB cells from wt and *bumble* mice were stimulated with 10 μg mL^−1^ anti‐IgM or 10 μg mL^−1^ LPS for 48 h and stained for TACI expression**.** Graph bars and error bars indicate mean ± s.d. Data are representative of two independent experiments with three mice in each group. Statistical significance was calculated using an unpaired Student's *t*‐test with ** and **** indicating *P* ≤ 0.01 and *P* ≤ 0.0001, respectively. **(d)** Mixed cultures of CD45.1 wt and CD45.2 *bumble* isolated splenic B cells were stimulated with 10 μg mL^−1^ anti‐IgM or 10 μg mL^−1^ LPS for 24 h and TACI levels were determined on wt *versus bumble* derived cells. Numbers adjacent to gates indicate frequencies. Data are shown from one experiment with 2 wt and 2 *bumble* mice. **(e)** TACI mRNA levels were determined by quantitative RT‐PCR. Expression of TACI mRNA transcripts is shown relative to Polr2a mRNA transcripts at steady‐state and 48 h after 10 μg mL^−1^ anti‐IgM or LPS stimulation of wt and *bumble* B cells. Data are representative of one experiment with 4 or 5 mice in each group. Statistical significance was calculated using the Mann–Whitney *U*‐test with * indicating *P* ≤ 0.05. **(f)** Soluble TACI was measured in serum of wt, *bumble* and TACI ^−/−^ mice. Data are representative of two independent experiments with 3 or 4 mice in each group. Error bars indicate mean ± s.d. Statistical significance was calculated using an unpaired Student's *t*‐test with *** indicating *P* ≤ 0.001. MFI, median fluorescence intensity.

Mice lacking TACI fail to respond to TI‐1 and TI‐2 antigens.^3^, ^5^ In *btk*‐deficient mice, the defect in TI‐2 responses was associated with defective TACI expression and function.^36^ In wt mice, steady‐state TACI levels are higher on MZB compared to FOB cells,^37^ which we also observed in our experiments. Notably, *bumble* MZB and FOB cells displayed no detectable surface TACI levels at steady‐state (Figure 3a). Since the MZB population in *bumble* is reduced in young mice and gradually increases upon aging, we performed these experiments in 6 to 7 months old wt and *bumble* mice.^24^ For some of the experiments, we used age‐matched TACI^−/−^ mice as controls.^5^ We also found that TACI expression was reduced on the transitional 1 B cell subset in the spleen (Supplementary figure 4a) and the mature B cell population in the bone marrow (Supplementary figure 4b). BCR and TLR stimulation is known to upregulate TACI expression on B cells.^37^ When examining cell surface TACI levels on *bumble* compared to wt B cells upon anti‐IgM stimulation (Figure 3b, upper panel), we found that *bumble* B cells were severely defective in this response. LPS stimulation resulted in upregulation of TACI on *bumble* B cells, although the frequencies of B cells expressing high TACI levels were lower in *bumble* B cell cultures compared to in wt B cell cultures (Figure 3b, lower panel). In contrast, upregulation of BAFFR and BCMA upon anti‐IgM or LPS stimulation was normal on *bumble* B cells (Supplementary figure 5). We also observed impaired TACI upregulation in sorted FOB cells, excluding the possibility that impaired TACI expression was due to the reduced MZB compartment in *bumble* mice (Figure 3c). Furthermore, impaired TACI upregulation was cell‐intrinsic, since in 1:1 mixed cultures of *bumble* and wt B cells, TACI upregulation was induced only on wt B cells (Figure 3d). In addition, TACI mRNA transcript levels were reduced in *bumble* compared to wt B cells upon both anti‐IgM and LPS stimulation (Figure 3e). TACI can be cleaved from the surface by the metalloproteinase ADAM10 to release soluble TACI (sTACI).^38^ We therefore investigated whether proteolytic cleavage caused low TACI expression on *bumble* B cells by measuring sTACI levels in the serum. We found significantly lower sTACI serum levels in *bumble* mice compared to in wt mice (Figure 3f), indicating that low TACI expression on *bumble* B cells was not the result of increased TACI cleavage. Similar to TLR4 stimulation, TLR9 ligation with CpG *in vitro* partially upregulated TACI on *bumble* B cells. Furthermore, CpG administration *in vivo* to *bumble* mice induced TACI expression on MZB cells (Supplementary figure 6a). It was previously demonstrated that CpG stimulation induced TACI expression and improved the response to TI‐2 antigens in *btk*‐deficient mice.^36^, ^39^ We found that *in vivo* co‐administration of CpG with NP‐Ficoll only minimally boosted NP‐specific antibody responses in *bumble* mice (Supplementary figure 6b). Taken together, *bumble* B cells lacked TACI expression at steady‐state and TACI upregulation was reduced compared to wt B cells upon BCR or TLR stimulation. However, *bumble* B cells were not completely unable to upregulate TACI, since stimulation via TLR4 or TLR9 resulted in a partial upregulation of TACI expression on these cells.

### B cells require IκBNS for a normal response to APRIL stimulation

**Figure 4:**
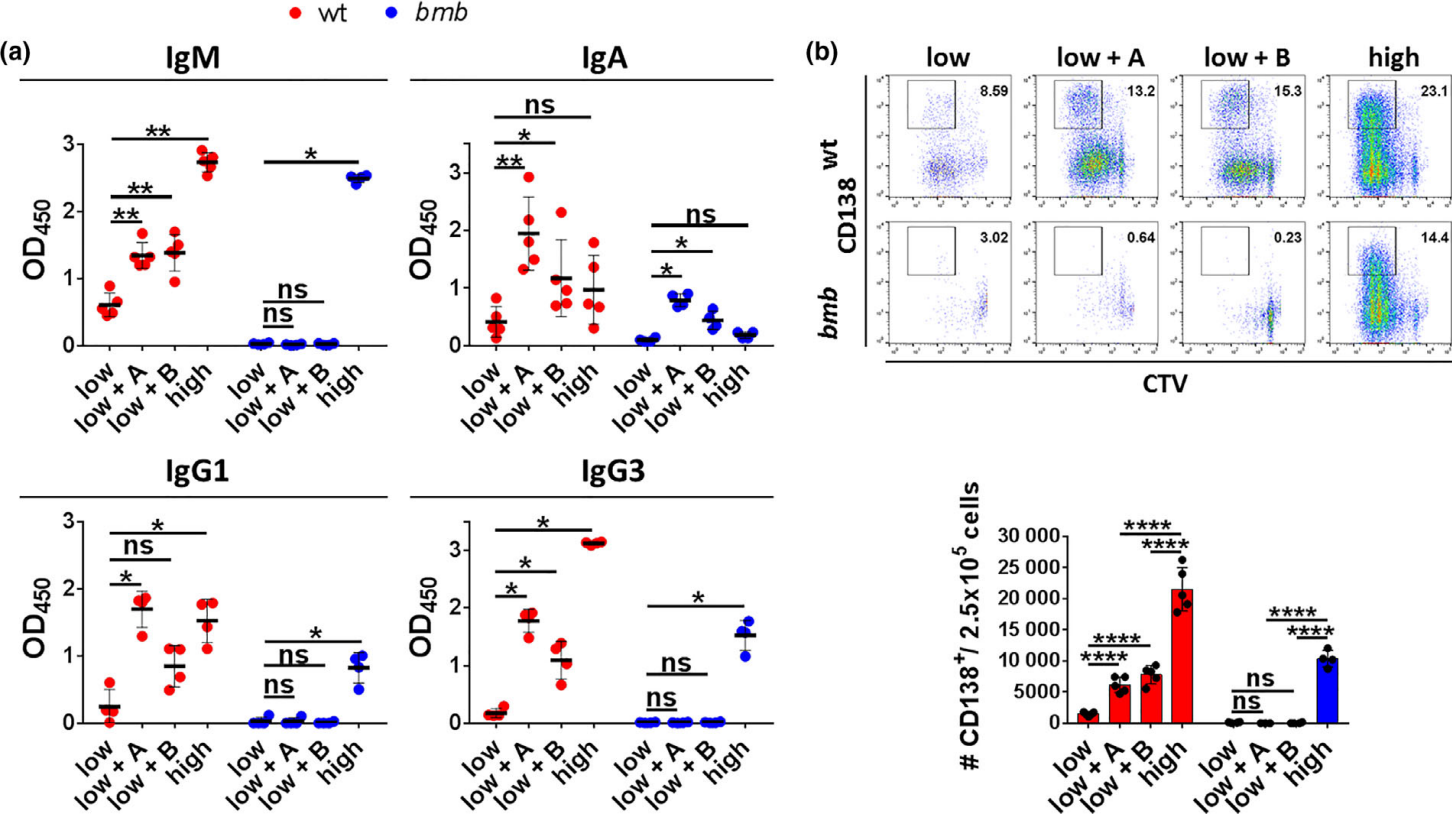
IκBNS is required for a normal response to the TACI ligands APRIL and BAFF. Isolated splenic B cells from wt and *bumble* mice were stimulated with a suboptimal dose of 100 ng mL^−1^ LPS (low), 100 ng mL^−1^ LPS and 1 μg mL^−1^ APRIL (low + A), 100 ng mL^−1^ LPS and 1 μg mL^−1^ BAFF (low + B), or 10 μg mL^−1^ LPS (high). **(a)** Levels of IgM, IgA, IgG1 and IgG3 were determined by ELISA from the supernatant of wt and *bumble* B cells after 6 days of stimulation. Error bars indicate mean ± s.d. Data are representative of two independent experiments with 4 or 5 mice in each group. Statistical significance was determined by the Mann–Whitney *U*‐test with ns, *, and ** indicating *P* > 0.05, *P* ≤ 0.01, and *P* ≤ 0.001, respectively. **(b)** Isolated splenic B cells from wt and *bumble* mice were labeled with CTV prior to stimulation and stained for CD138 expression at 84 h. Representative plots indicate cells undergoing PC differentiation (upper panel). Numbers adjacent to gates indicate cell frequencies. Total number of CD138^+^ cells per 250.000 seeded B cells (lower panel). Bars and error bars indicate mean ± s.d. Data are representative of two independent experiments with 4 or 5 mice in each group. Statistical significance was determined by an unpaired Student's *t*‐test with ns and **** indicating *P* > 0.05 and *P* ≤ 0.0001, respectively.

It was previously shown that APRIL synergizes with LPS in promoting antibody secretion and class‐switching by signaling through TACI.^4^ To study the effect of impaired TACI expression in IκBNS‐deficient mice in response to TACI ligands, we stimulated wt and *bumble* B cells *in vitro* with APRIL or BAFF together with the suboptimal concentration of LPS (100 ng mL^−1^). We determined the levels of IgM, IgG1, IgG3 and IgA at day 6. Co‐stimulation with APRIL or BAFF both enhanced secretion of IgM, IgA, IgG1 and IgG3 to the low LPS dose from wt B cells. However, *bumble* B cells were completely unresponsive to TACI ligation as the secretion of IgM, IgG1 and IgG3 was not detectable in the cultures with low LPS regardless of the presence of APRIL or BAFF (Figure 4a). In contrast, only the high concentration of LPS (10 μg mL^−1^) induced IgM, IgG1 and IgG3 secretion from *bumble* B cells, although at reduced levels compared to those from wt B cells. In terms of IgA secretion, a moderate increase was detected in *bumble* B cell cultures co‐stimulated with APRIL or BAFF, which is likely to be attributed to normal expression of BCMA.^4^ In wt B cell cultures, APRIL or BAFF addition to the suboptimal concentration of LPS both increased the frequencies of live (L/D far red^−^) cells at 84 h (3.5 days) (Supplementary figure 7a), consistent with previous reports that TACI ligation provides a survival signal.^40^ In *bumble* B cell cultures, we also observed an increase in viability (Supplementary figure 7a), but both frequencies and numbers were moderately reduced compared to wt in response to the low and high concentration of LPS (Supplementary figure 7c). Addition of APRIL or BAFF to the low concentration of LPS in both wt and *bumble* cultures increased the frequencies of blasted cells modestly but the difference was not significant (Supplementary figure 7b). Consistent with previous findings that APRIL enhances PC generation,^4^ we observed a significant increase in the total numbers of CD138^+^ cells in the presence of APRIL but also BAFF in wt cultures (Figure 4b). However, in *bumble* B cell cultures, CD138^+^ cells were observed only in response to the high LPS concentration (10 μg mL^−1^) (Figure 4b). Thus, *bumble* B cells did not respond to TACI ligation even when cells were stimulated with LPS, consistent with low TACI expression on the cell surface under these conditions (Figure 3b, Supplementary figure 6a).

### IκBNS‐deficient B cells fail to normally regulate transcription factors involved in PC differentiation in response to LPS stimulation

**Figure 5:**
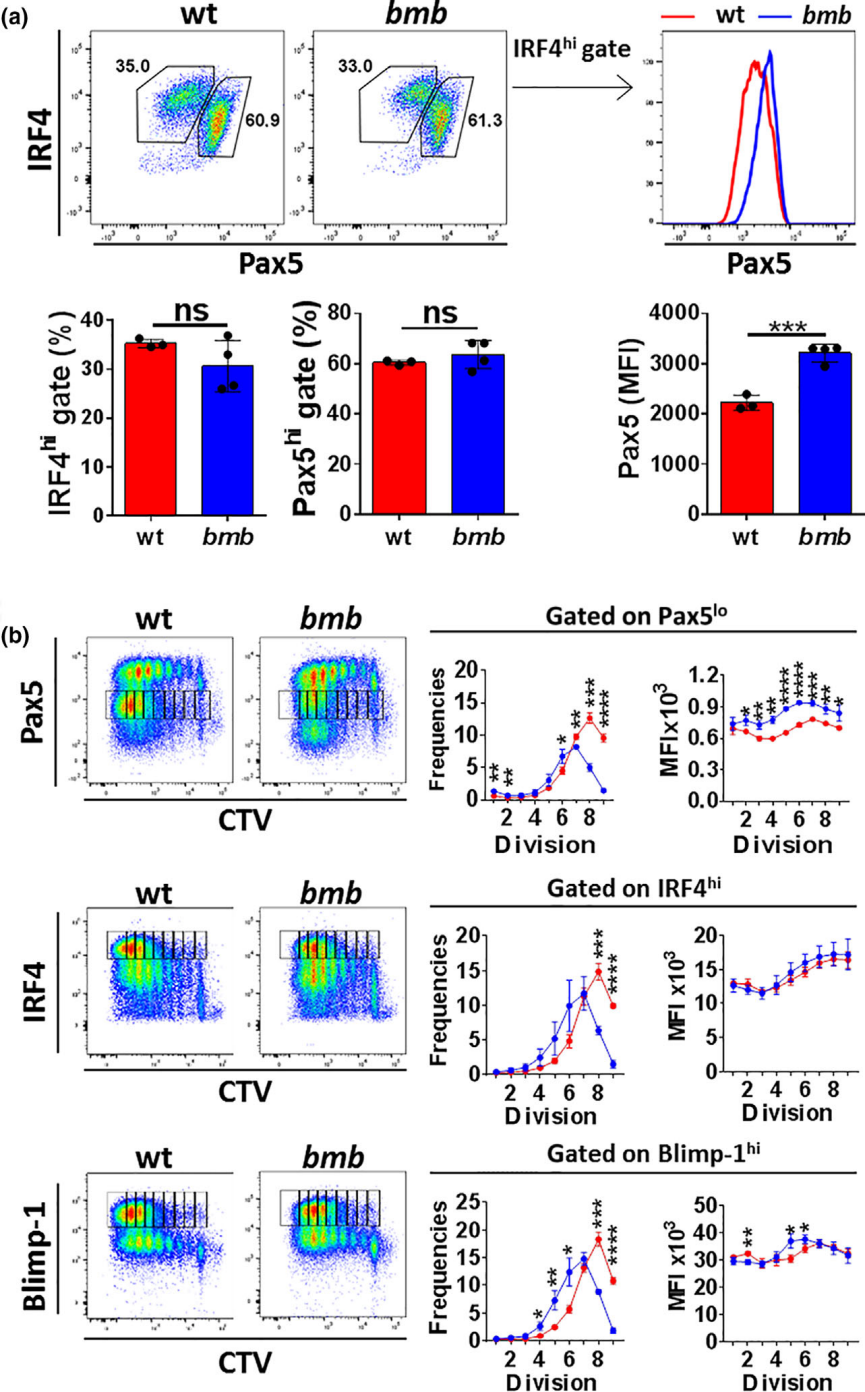
IκBNS is required for intact transcriptional regulation of PC differentiation. Isolated splenic B cells from wt and *bumble* mice were stimulated with 10 μg mL^−1^ LPS. **(a)** Intracellular expression of the transcription factors IRF4 and Pax5 at 84 h. Representative plots show activated B cells diverging into Pax5^lo^ IRF4^hi^ and Pax5^hi^ IRF4^int^ populations (upper left panel). Numbers adjacent to gates indicate frequencies of cells within the gate. Frequencies of the Pax5^lo^ IRF4^hi^ and Pax5^hi^ IRF4^int^ populations with error bars indicating mean ± s.d. (lower left panel). Representative histogram showing Pax5 expression within the IRF4^hi^ population (upper right panel). Expression of Pax5 in wt and *bumble* cells at 84 h after LPS stimulation (lower right panel). Data are representative of four independent experiments with 3 or 4 mice in each group. Statistical significance was determined by the Mann–Whitney *U*‐test with ns and *** indicating *P* > 0.05 and *P* ≤ 0.001, respectively. **(b)** Isolated splenic B cells from wt and *bumble* mice were CTV‐labeled and stained for Pax5, IRF4 and Blimp‐1 at 84 h poststimulation. Representative plots are shown with gating for the Pax5^lo^, IRF4^hi^ and Blimp‐1^hi^ populations per individual division cycle (left panels). Frequencies and corresponding MFI values are shown for the Pax5^lo^, IRF4^hi^ and Blimp‐1^hi^ populations per individual division cycle as gated on in the FACS plots (middle panels and right panels). Data are representative of three independent experiments with 3 or 4 mice in each group. Error bars indicate mean ± s.d. Statistical significance was determined by unpaired *t*‐test with *, **, *** and **** indicating *P* ≤ 0.05, *P* ≤ 0.01, *P* ≤ 0.001, *P* ≤ 0.0001, respectively. MFI, median fluorescent intensity. Statistical test used to determine significance.

As *bumble* mice were unable to establish intact LPS‐induced plasmablast and PC compartments, we next examined the PC differentiation program in more detail. B cell differentiation is regulated in a dose‐dependent manner by the transcription factor IRF4, which initiates PC generation when expressed at high concentrations 41, 42 by controlling Blimp‐1 expression.^43^ In addition, efficient terminal PC differentiation coincides with downregulation of Pax5, a key transcription factor for maintenance of the B cell lineage, through direct repression by Blimp‐1.^44^, ^45^ Activated B cells separate into a population of Pax5^lo^IRF4^hi^ cells, which are committed to PC differentiation, and a population of Pax5^hi^IRF4^int^ cells, which preserve a germinal center B cell phenotype.^46^ At 84 h (3.5 days) of LPS stimulation, both the Pax5^lo^IRF4^hi^ and the Pax5^hi^IRF4^int^ populations were present in *bumble* and TACI‐deficient B cell cultures similar to wt B cells (Figure 5a, left panel, Supplementary figure 8). However, *bumble* B cells, unlike wt and TACI ^−/−^ B cells, were unable to fully downregulate Pax5 in the IRF4^hi^ population (Figure 5a, right panel, Supplementary figure 8). As PC differentiation is a division‐linked process, downregulation of Pax5 and upregulation of IRF4 and Blimp‐1 coincide with cellular divisions.^46^ Therefore, we examined Pax5, IRF4 and Blimp‐1 expression in LPS‐induced cellular divisions of wt and *bumble* B cells. Frequencies of Pax5^lo^, IRF4^hi^ and Blimp‐1^hi^ cells in *bumble* B cells were all significantly reduced in the late divisions (division 7+/8+, respectively) compared to in wt B cells (Figure 5b). Expression levels of Pax5, as measured by median fluorescent intensity (MFI), were consistently increased in the Pax5^hi^ population in *bumble* B cells throughout all divisions, whereas expression of IRF4 in the IRF4^hi^ population was unaffected (Figure 5b). Although the frequencies of Blimp‐1^hi^ cells in *bumble* B cells were significantly reduced compared to in wt B cells in the late divisions, they were increased in the intermediate divisions (divisions 4–6) and displayed elevated Blimp‐1 expression in divisions 5 and 6 (Figure 5b). Thus, *bumble* B cells were unable to completely suppress Pax5 in the presence of Blimp‐1, and generated fewer Pax5^lo^, IRF4^hi^ and Blimp‐1^hi^ cells in the late divisions. These results demonstrated that IκBNS is required for intact transcriptional regulation during PC development.

## Discussion

The TI antigens are generally polysaccharides derived from bacterial capsules that engage the BCR and/or TLRs, which both activate the NF‐κB pathway. We have previously shown that antibody responses to the TI antigens NP‐Ficoll and Pneumococcal polysaccharides (Pneumovax) are impaired in *bumble* mice.^22^, ^25^ These mice lack expression of IκBNS, a nuclear regulator of the NF‐κB pathway. However, the role of IκBNS in the generation of TI antibody responses was not addressed in depth previously. In this study, we show that B cells lacking functional IκBNS display reduced proliferation, TACI upregulation and responsiveness to TACI ligands, and that terminal PC differentiation is impaired at the transcriptional level.

Several studies have underlined the importance of TACI signaling for TI antibody responses.^3^, ^4^, ^5^ B cells express surface TACI from transitional to mature stages, with MZB and B‐1 cells displaying the highest levels.^37^, ^47^, ^48^ TACI is upregulated upon stimulation via the BCR, TLR4 and TLR9.^37^, ^47^ We demonstrate here that IκBNS is essential in these processes, as *bumble* B cells lacked normal TACI expression and failed to upregulate TACI after BCR or TLR stimulation. *btk‐*deficient mice display impaired TACI expression, consistent with a direct role of BCR signaling for maintaining surface TACI.^3^ Interestingly, in contrast to *btk*‐deficient B cells, the early activation of IκBNS‐deficient *bumble* B cells, other than reduced TACI expression, was similar to that observed in wt B cells in response to anti‐IgM and LPS.^18^, ^19^ B cells lacking another component of NF‐κB signaling, Hoip‐1 of the LUBAC complex, are also activated normally upon BCR stimulation, yet fail to respond to TI‐2 antigens.^49^ Interestingly, LUBAC was not required for TACI upregulation upon BCR stimulation, but for TACI‐induced NF‐κB activation by APRIL.^49^ Impaired responses in *bumble* to TACI ligands could be due to altered NF‐κB regulation in the absence of IκBNS in addition to attenuated TACI expression. Enhancement of antibody responses to low doses of LPS by APRIL and BAFF is relevant in the context of physiological quantities of pathogens and antigens upon infection, emphasizing the importance of intact TACI expression and signaling. This highlights different mechanisms behind impaired response to TI‐2 antigens in various NF‐κB deficient strains, illustrating the complexity of this pathway. Moreover, as the NF‐κB1 and NF‐κB2 proteins were linked to the CVID phenotype in patients,^10^, ^11^, ^12^, ^13^ this emphasizes the possibility that defects in various components of the NF‐κB pathway could contribute to pathogenesis of immunodeficiencies in humans.

Similar to TACI^−/−^ mice, *bumble* mice displayed reduced serum IgM,^18^ impaired Ig secretion to APRIL stimulation, and reduced PC frequencies in response to TI antigens. However, PC differentiation in *bumble* B cells was more severely impaired compared to in TACI^−/−^ B cells. PC differentiation requires a sequence of ordered events including upregulating IRF4 and downregulating Pax5, which precedes upregulation of Blimp‐1 and CD138 expression.^50^ TACI has been suggested to be involved in maintenance of Blimp‐1 expression during PC generation.^51^ Interestingly, our data showed normal IRF4 expression and Pax5 downregulation in TACI^−/−^ B cells, indicating that PC differentiation was unaffected. Therefore, the defective TI response in TACI^−/−^ mice could be due to a requirement for TACI‐dependent Blimp‐1 expression for PC maintenance rather than differentiation.^51^ A previous study showed reduced mRNA levels of IRF4 and Blimp‐1 in LPS‐activated B cells derived from IκBNS^−/−^ mice.^19^ We found that B cells from *bumble* mice were able to express IRF4 and Blimp‐1 at the protein level in response to LPS, however, with reduced frequencies of IRF4^hi^ and Blimp‐1^hi^ cells at the later stages of division. The reduced proliferation caused by the loss of IκBNS likely results in fewer cells at the commitment stage for PC differentiation, which could explain the decrease in mRNA transcripts for PC markers in total B cell cultures. Additionally, despite normal expression of IRF4 and enhanced initial induction of Blimp‐1, Pax5 was not fully suppressed in *bumble* B cells. We found that Pax5^lo^, IRF4^hi^ and Blimp‐1^hi^ B cells were lost after division 6 which coincides with commitment to terminal PC differentiation. Hence, in *bumble* mice, PC generation appears to be impaired at the terminal PC differentiation phase where we observed incomplete downregulation of Pax5. Pax5 expression in B cells is regulated by an enhancer containing NF‐κB‐binding regions.^52^ Thus, it is possible that IκBNS modulates NF‐κB activity at this enhancer.

**Figure 6:**
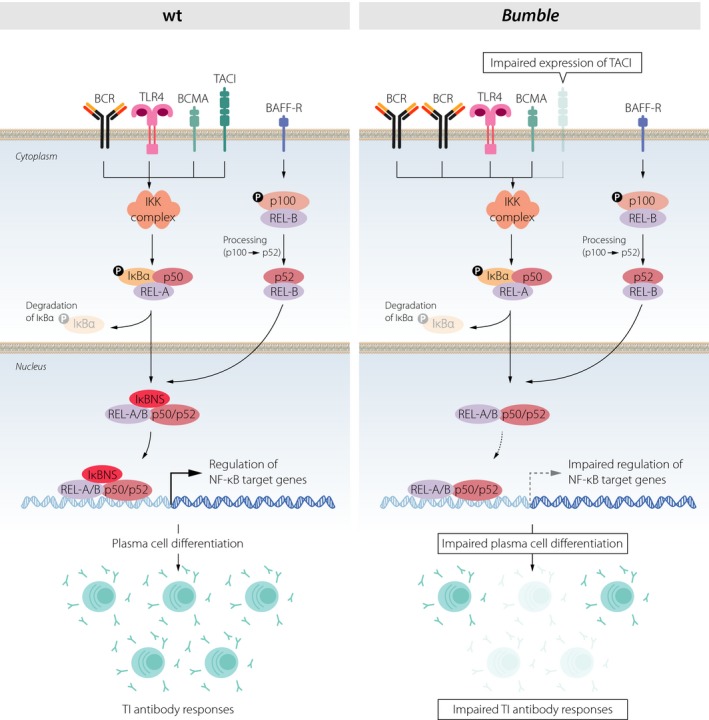
Schematic representation of how ablation of IκBNS affects B cell activation and PC differentiation. Activation of B cells in response to TI antigens is initiated through ligation of the BCR and/or Toll‐like receptors, and is enhanced through TACI. These surface receptors all trigger activation of the classical NF‐κB pathway by degrading the inhibitory IκB protein, consequently releasing the NF‐κB proteins that translocate to the nucleus where they are transcriptionally active. In *bumble*, the transcriptional regulation of NF‐κB target genes is altered in the absence of IκBNS, resulting in impaired TACI expression, PC differentiation and TI antibody responses.

In conclusion, our results demonstrate that IκBNS is required for TACI upregulation, responsiveness to both APRIL and BAFF, proliferation and intact differentiation of B cells into antibody‐secreting PC, all of which are required for robust TI antibody responses (Figure 6). The results reported in this study help explain mechanisms for the lack of response to TI antigens associated with defects in the different components of NF‐κB signaling, which may have bearing on some cases of CVID.

## Methods

### Mice

Mice were maintained at the animal research facilities, MTC, and KM‐W, at Karolinska Institutet. Studies were performed in accordance with institutionally approved protocols and Committee for Animal Ethics (Stockholms Norra Djurförsöksetiska nämnd) approval. For bone marrow chimera experiments, C57BL/6J CD45.2 and CD45.1 mice were purchased from the Jackson laboratory. Spleens were obtained from TACI ^−/−^ mice as described previously.^5^ Mice harboring the *bumble* mutation in the gene encoding IκBNS were described previously.^18^, ^22^

### Cell preparation

Splenocytes were prepared as single cell suspensions using 70 μm cell strainers in RPMI 1640 (HyClone, Logan, UT) supplemented with 2 mm l‐glutamine, penicillin (100 IU)‐streptomycin (100 μg mL^−1^) (Sigma‐Aldrich, St Louis, MO), β‐mercaptoethanol (0.05 mm) (Life Technologies, Waltham, MA) and 10% fetal bovine serum (HyClone) (complete RPMI medium). Splenocyte and bone marrow cell suspensions were washed once in Ca^2+^‐ and Mg^2+^‐free PBS (Sigma‐Aldrich) and treated with red blood cell lysis buffer before further processing.

### *In vitro* B cell cultures

Mouse B cells were isolated by the EasySep Mouse B cell Negative selection Kit (STEMCELL Technologies, Vancouver) according to the manufacturer's protocol. Purity based on CD19 expression was around 95–97% as determined by flow cytometry. Cells were seeded at a cell density of 2.5 x 10^5^ or 3 x 10^6 ^mL^−1^, for flow cytometry staining and ELISA, and RNA extraction, respectively. Cells were stimulated in complete RPMI with 10 μg mL^−1^ of unconjugated goat anti‐mouse IgM F(ab’)_2_ (Jackson ImmunoResearch Laboratories, West Grove, PA), 10 μg mL^−1^ or 100 ng mL^−1^ lipopolysaccharide (LPS) from *E. coli* 0111:B4 (Sigma‐Aldrich), 1 μg mL^−1^ APRIL (R&D Systems, Minneapolis, MN), 1 μg mL^−1^ BAFF (R&D Systems), or 1 μg mL^−1^ CpG oligonucleotides ODN 1826 (InvivoGen, San Diego, CA). To induce IkBα degradation, 1 x 10^6 ^cells were stimulated in 100 μL complete RPMI medium for 90 min with 10 μg mL^−1^ of unconjugated goat anti‐mouse IgM F(ab’)_2_ (Jackson ImmunoResearch Laboratories), 50 ng mL^−1^ PMA and 1 μm ionomycin, or 0.1 μm calyculin A, in the presence of 10 μm cyclohexamide (all from Sigma‐Aldrich).

### Immunization

Mice were immunized intraperitoneally (i.p.) with 50 μg NP^40^‐Ficoll (Biosearch Technologies, Novato, CA) in 200 μL PBS. CpG oligonucleotides ODN 1826 (InvivoGen) were administered at a dose of 50 μg in 200 μL i.p. LPS of the *Salmonella Minnesota* strain (Enzo Life Sciences, Farmingdale, NY) was injected intravenously (i.v.) at a dose of 5 μg in 100 μL PBS.

### Real‐time PCR

RNA was isolated from 3 × 10^6^ B cells using Trizol (Invitrogen) followed by DNase‐treatment using TURBO DNA‐free kit (Thermo Fisher Scientific, Waltham, MA) according to the manufacturer's instructions. RNA concentration was measured on Qubit (Thermo Fisher Scientific). cDNA synthesis was performed with 100 ng of RNA using SuperScript IV (Invitrogen) according to the manufacturer's instructions. Real‐time PCR was prepared with 1 μL of cDNA and 1 μm of the *forward* and *reverse* primer in RT^2^ SYBR Green Master Mix (Bio‐Rad Laboratiers, Hercules, CA) in a total volume of 10 μL. Primers used for amplification were TACI‐forward 5’‐ATGGTCGTAGTACCTGCCTTG‐3’, TACI‐reverse 5’‐ATGGCATTCTGCCCCAAAGAT‐3’ (reference 4), Polr2a‐forward 5’‐CGGTTGAATCTTAGTGTGAC‐3’, and Polr2 a‐reverse 5’‐ATAGCCAACTCTTGGATCTC‐3’ (reference 53). Assays were performed in 384‐well plates on Bio‐Rad CFX384 thermal cycler under the following conditions; denaturation at 95°C for 2 min, PCR amplification at 95°C for 5 s and extension at 60°C for 20 s for 45 cycli, followed by melt‐curve analysis of 0.5°C increments per 5 s from 65 to 95°C.

### ELISA

ELISA plates (Nalge Nunc, Rochester, NY) were coated with 500 ng/well of NP^25^ conjugated with BSA (Biosearch Technologies). To detect IgM, IgG1, IgG3 or IgA, plates were coated with unconjugated goat anti‐mouse IgM (Southern Biotech, , Birmingham, AL), IgG (Southern Biotech), or IgA (BD, Franklin Lakes, NJ), respectively. After incubation overnight (4°C), washing with PBS + 0.05% Tween20 and blocking for 1 h with PBS containing 2% dry milk, 50 μL of culture supernatant was added in a total volume of 150 μL, followed by threefold serial dilutions in blocking buffer and incubated for 2 h at room temperature (RT). Plates were washed six times, and primary antibodies, biotinylated goat anti‐mouse IgM, HRP‐coupled anti‐IgG1, HRP‐coupled anti‐IgG3 (Southern Biotech, Birmingham, AL), or biotinylated goat anti‐mouse IgA (BD Franklin Lakes, NJ), were added in 100 μL PBS/well followed by incubation for 1.5 h, at RT. After six washes, streptavidin‐HRP was added to biotinylated antibodies in 100 μL PBS/well and incubated for 1 h at RT. The assay was developed with TMB substrate (KPL), the reaction was stopped with 1 m H_2_SO_4_, and the OD was read at 450 nm using an Asys Expert 96 ELISA reader (Biochrom Ltd, Cambridge, UK). For detection of soluble TACI, the mouse TACI/TNFRSF13B DuoSet ELISA kit (R&D Systems) was used, according to the manufacturer's instructions.

### Flow cytometry and cell sorts

To block nonspecific binding to Fc receptors, cells were incubated with anti‐CD16/32 antibody (BD Franklin Lakes), and then stained with different panels of fluorochrome conjugated monoclonal antibodies (Supplementary table 1) in PBS/2% FBS. For intracellular staining of transcription factors, cells were fixed, permeabilized and stained using the transcription factor buffer set (BD Franklin Lakes) according to the manufacturer's instructions. To track divisions, CellTrace Violet (Invitrogen) was used to label cells according to the manufacturer's protocol. Samples were run using a BD LSR II, BD Fortessa, BD Verse or FACSCalibur machine and data were analyzed by FlowJo software v9.6.4 (Tree Star, Ashland, OR). For follicular B cell sorts, splenocytes were stained with LIVE/DEAD Aqua dye, B220, CD21/35 and CD23 antibodies and sorted on the FACSAria II cell sorter (BD Franklin Lakes).

### Statistics

Differences between groups were analyzed by a Student's *t*‐test or Mann–Whitney *U‐*test (GraphPad Prism v6.0f). Statistical significance is indicated with ns for *P *>* *0.05, * for *P *≤* *0.05, ** for *P *≤* *0.01, *** for *P *≤* *0.001 and **** for *P* ≤ 0.0001.

## Conflict of Interest

The authors have no conflicting financial interests.
