# Supplementary material for: TACI expression and plasma cell differentiation are impaired in the absence of functional IκBNS
Source: Immunol Cell Biol. 2019 Jan 30;97(5):485–97. doi: 10.1111/imcb.12228 (PMC6850186; doi:10.1111/imcb.12228)
Supplement: Supplementary file 1 [file IMCB-97-485-s001.pptx]

## Slide 1
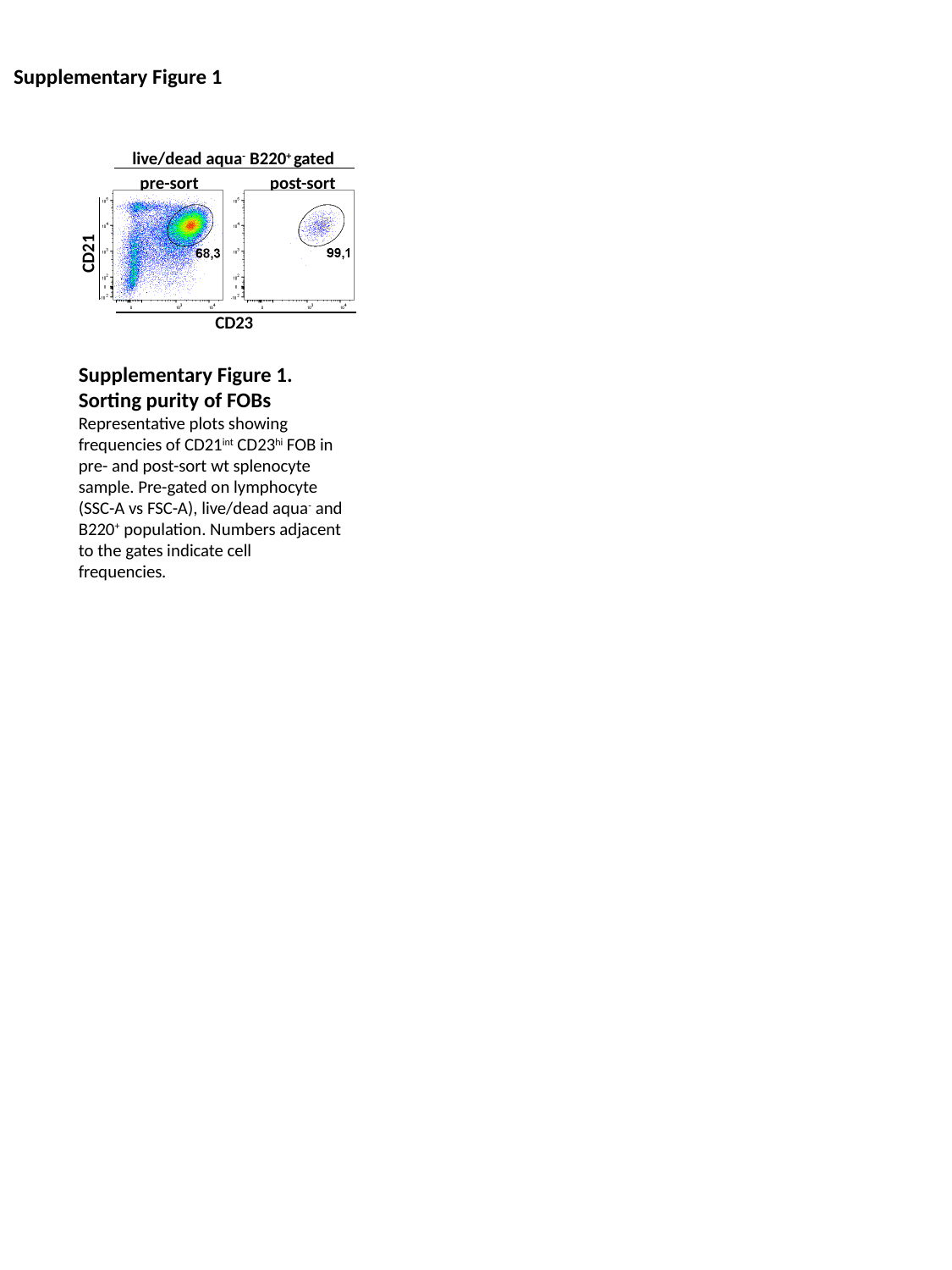

Supplementary Figure 1
live/dead aqua- B220+ gated
pre-sort
post-sort
CD21
CD23
Supplementary Figure 1. Sorting purity of FOBs
Representative plots showing frequencies of CD21int CD23hi FOB in pre- and post-sort wt splenocyte sample. Pre-gated on lymphocyte (SSC-A vs FSC-A), live/dead aqua- and B220+ population. Numbers adjacent to the gates indicate cell frequencies.

## Slide 2
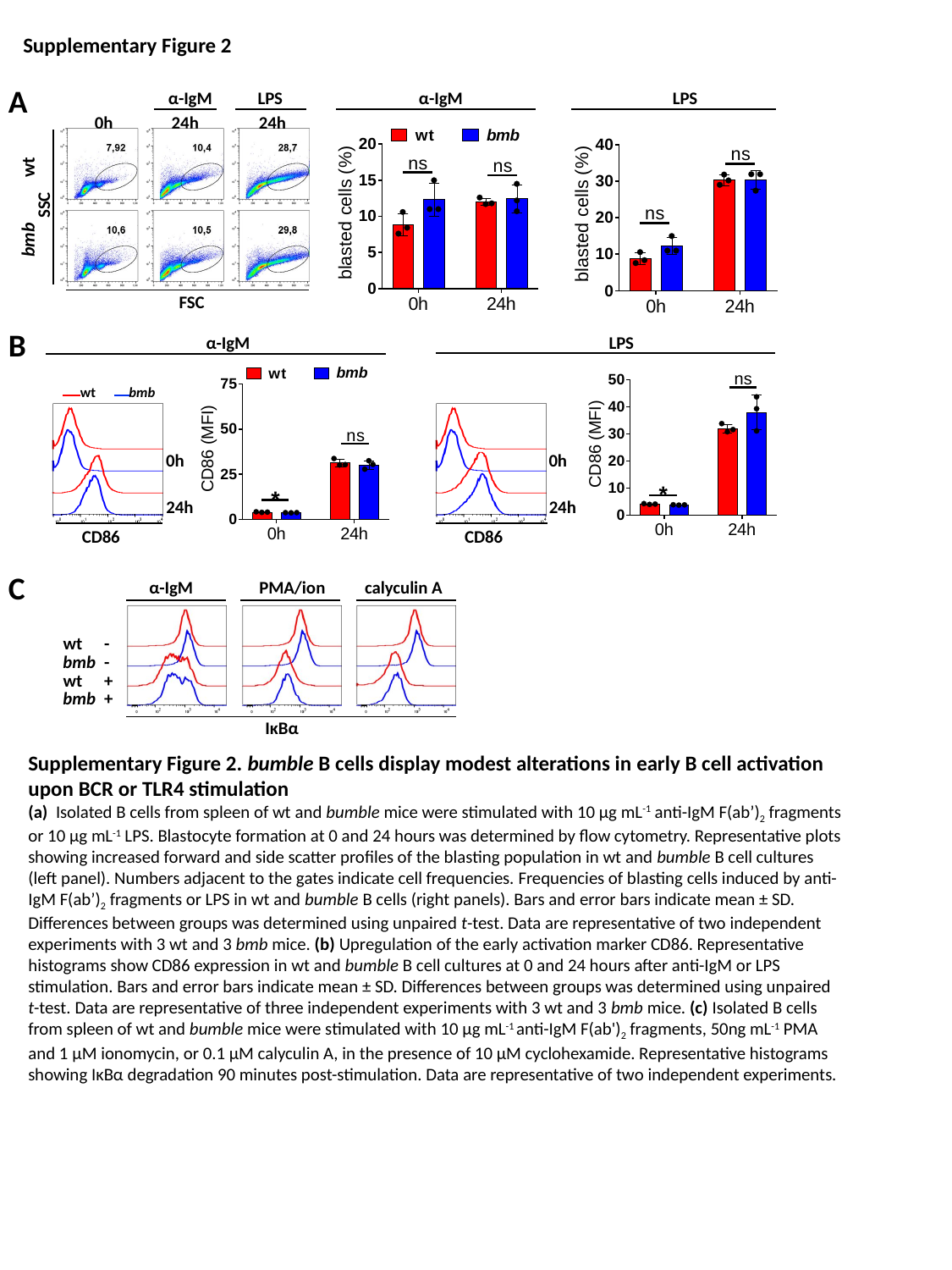

Supplementary Figure 2
A
α-IgM
LPS
α-IgM
LPS
0h
24h
24h
wt
SSC
bmb
FSC
B
LPS
α-IgM
wt
bmb
0h
0h
24h
24h
CD86
CD86
C
α-IgM
PMA/ion
calyculin A
wt
-
bmb
-
wt
+
bmb
+
IκBα
Supplementary Figure 2. bumble B cells display modest alterations in early B cell activation upon BCR or TLR4 stimulation
(a) Isolated B cells from spleen of wt and bumble mice were stimulated with 10 μg mL-1 anti-IgM F(ab’)2 fragments or 10 μg mL-1 LPS. Blastocyte formation at 0 and 24 hours was determined by flow cytometry. Representative plots showing increased forward and side scatter profiles of the blasting population in wt and bumble B cell cultures (left panel). Numbers adjacent to the gates indicate cell frequencies. Frequencies of blasting cells induced by anti-IgM F(ab’)2 fragments or LPS in wt and bumble B cells (right panels). Bars and error bars indicate mean ± SD. Differences between groups was determined using unpaired t-test. Data are representative of two independent experiments with 3 wt and 3 bmb mice. (b) Upregulation of the early activation marker CD86. Representative histograms show CD86 expression in wt and bumble B cell cultures at 0 and 24 hours after anti-IgM or LPS stimulation. Bars and error bars indicate mean ± SD. Differences between groups was determined using unpaired t-test. Data are representative of three independent experiments with 3 wt and 3 bmb mice. (c) Isolated B cells from spleen of wt and bumble mice were stimulated with 10 μg mL-1 anti-IgM F(ab')2 fragments, 50ng mL-1 PMA and 1 μM ionomycin, or 0.1 μM calyculin A, in the presence of 10 μM cyclohexamide. Representative histograms showing IκBα degradation 90 minutes post-stimulation. Data are representative of two independent experiments.

## Slide 3
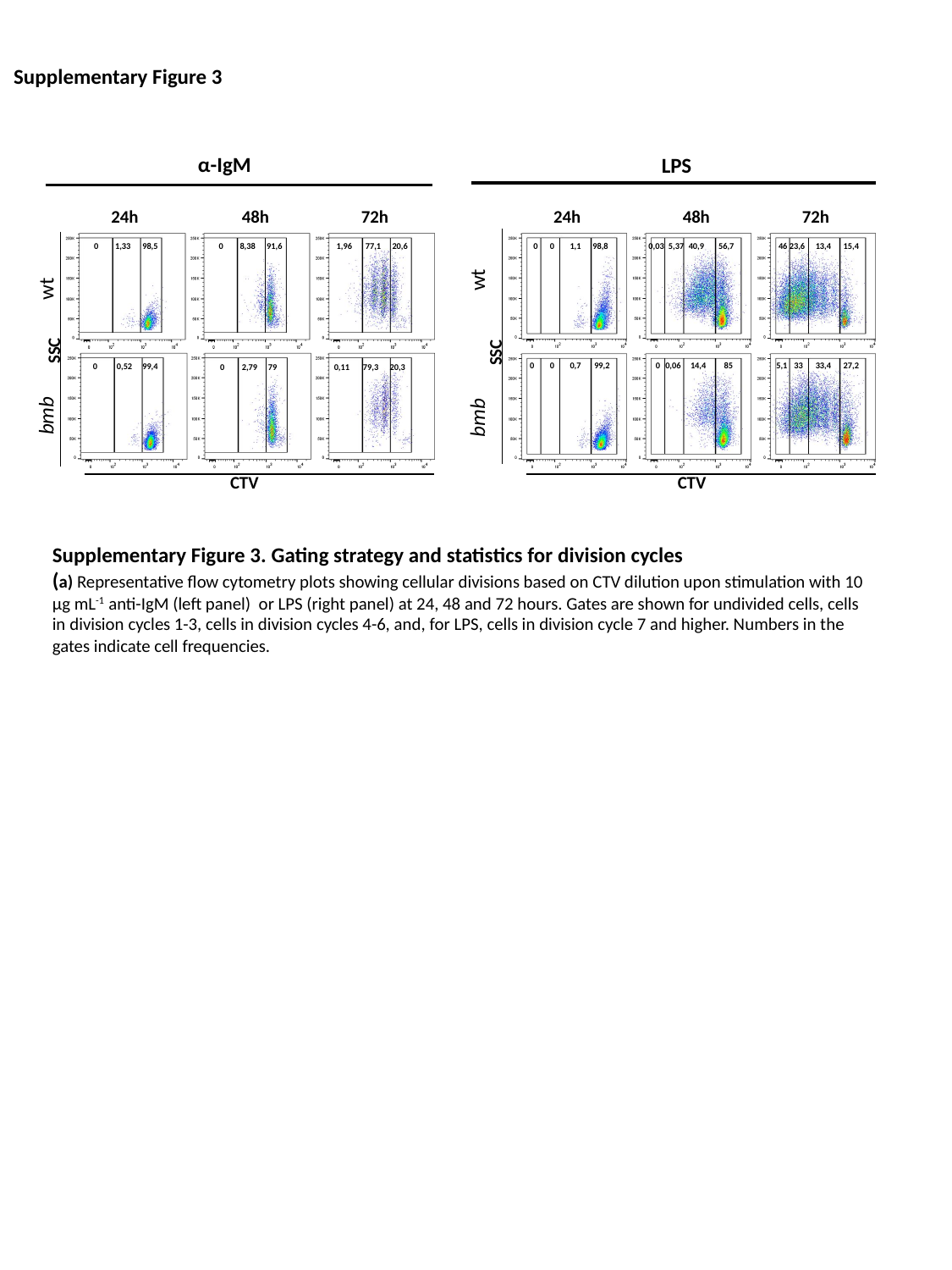

Supplementary Figure 3
α-IgM
LPS
24h
48h
72h
24h
48h
72h
0
1,33
98,5
0
8,38
91,6
1,96
77,1
20,6
0
0
1,1
98,8
0,03
5,37
40,9
56,7
46
23,6
13,4
15,4
wt
wt
SSC
SSC
0
0
0,7
99,2
0
0,06
14,4
85
5,1
33
33,4
27,2
0
0,52
99,4
0
2,79
79
0,11
79,3
20,3
bmb
bmb
CTV
CTV
Supplementary Figure 3. Gating strategy and statistics for division cycles
(a) Representative flow cytometry plots showing cellular divisions based on CTV dilution upon stimulation with 10 μg mL-1 anti-IgM (left panel) or LPS (right panel) at 24, 48 and 72 hours. Gates are shown for undivided cells, cells in division cycles 1-3, cells in division cycles 4-6, and, for LPS, cells in division cycle 7 and higher. Numbers in the gates indicate cell frequencies.

## Slide 4
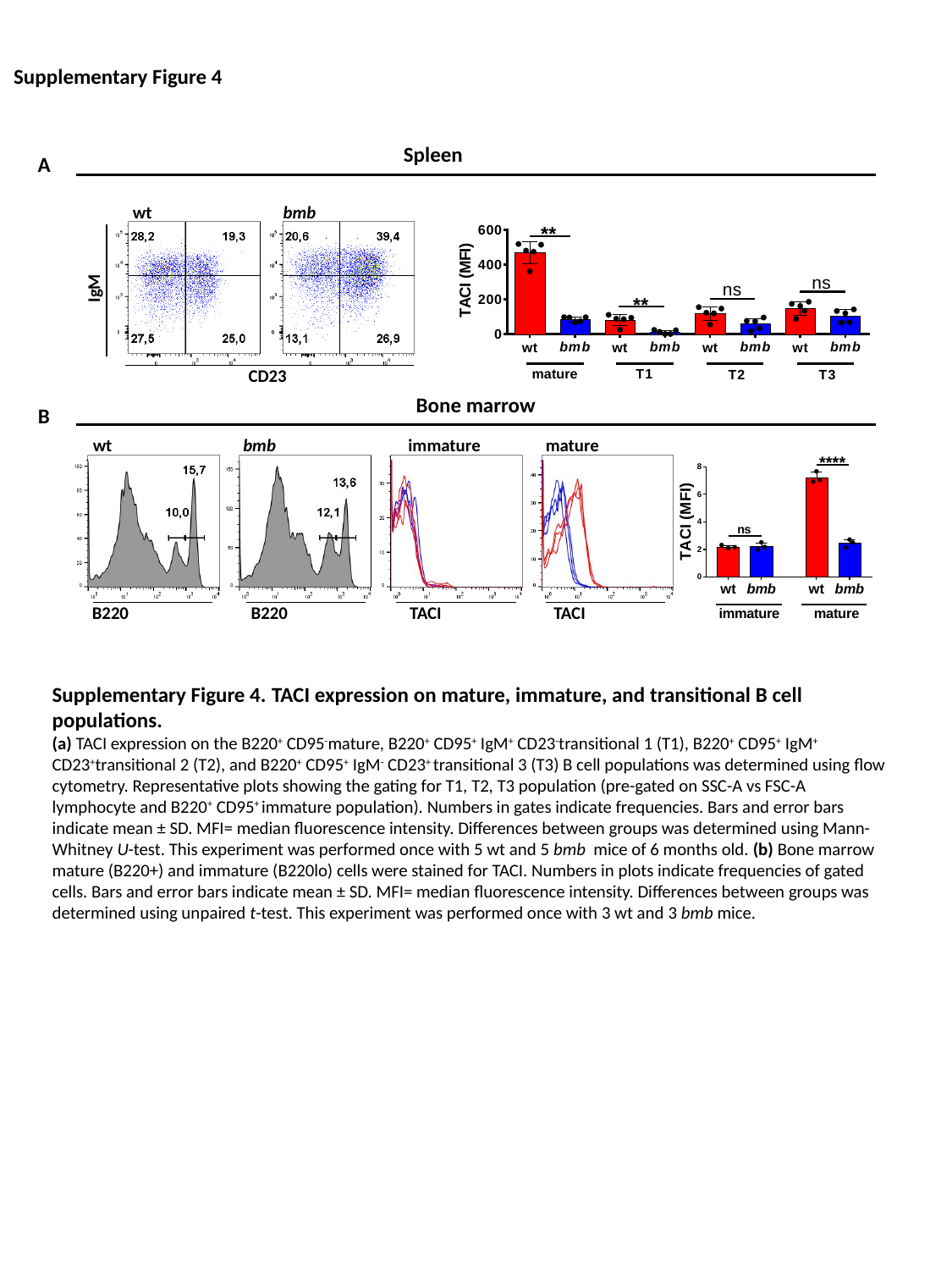

Supplementary Figure 4
Spleen
A
wt
bmb
IgM
CD23
Bone marrow
B
wt
bmb
immature
mature
B220
B220
TACI
TACI
Supplementary Figure 4. TACI expression on mature, immature, and transitional B cell populations.
(a) TACI expression on the B220+ CD95-mature, B220+ CD95+ IgM+ CD23-transitional 1 (T1), B220+ CD95+ IgM+ CD23+transitional 2 (T2), and B220+ CD95+ IgM- CD23+ transitional 3 (T3) B cell populations was determined using flow cytometry. Representative plots showing the gating for T1, T2, T3 population (pre-gated on SSC-A vs FSC-A lymphocyte and B220+ CD95+ immature population). Numbers in gates indicate frequencies. Bars and error bars indicate mean ± SD. MFI= median fluorescence intensity. Differences between groups was determined using Mann-Whitney U-test. This experiment was performed once with 5 wt and 5 bmb mice of 6 months old. (b) Bone marrow mature (B220+) and immature (B220lo) cells were stained for TACI. Numbers in plots indicate frequencies of gated cells. Bars and error bars indicate mean ± SD. MFI= median fluorescence intensity. Differences between groups was determined using unpaired t-test. This experiment was performed once with 3 wt and 3 bmb mice.

## Slide 5
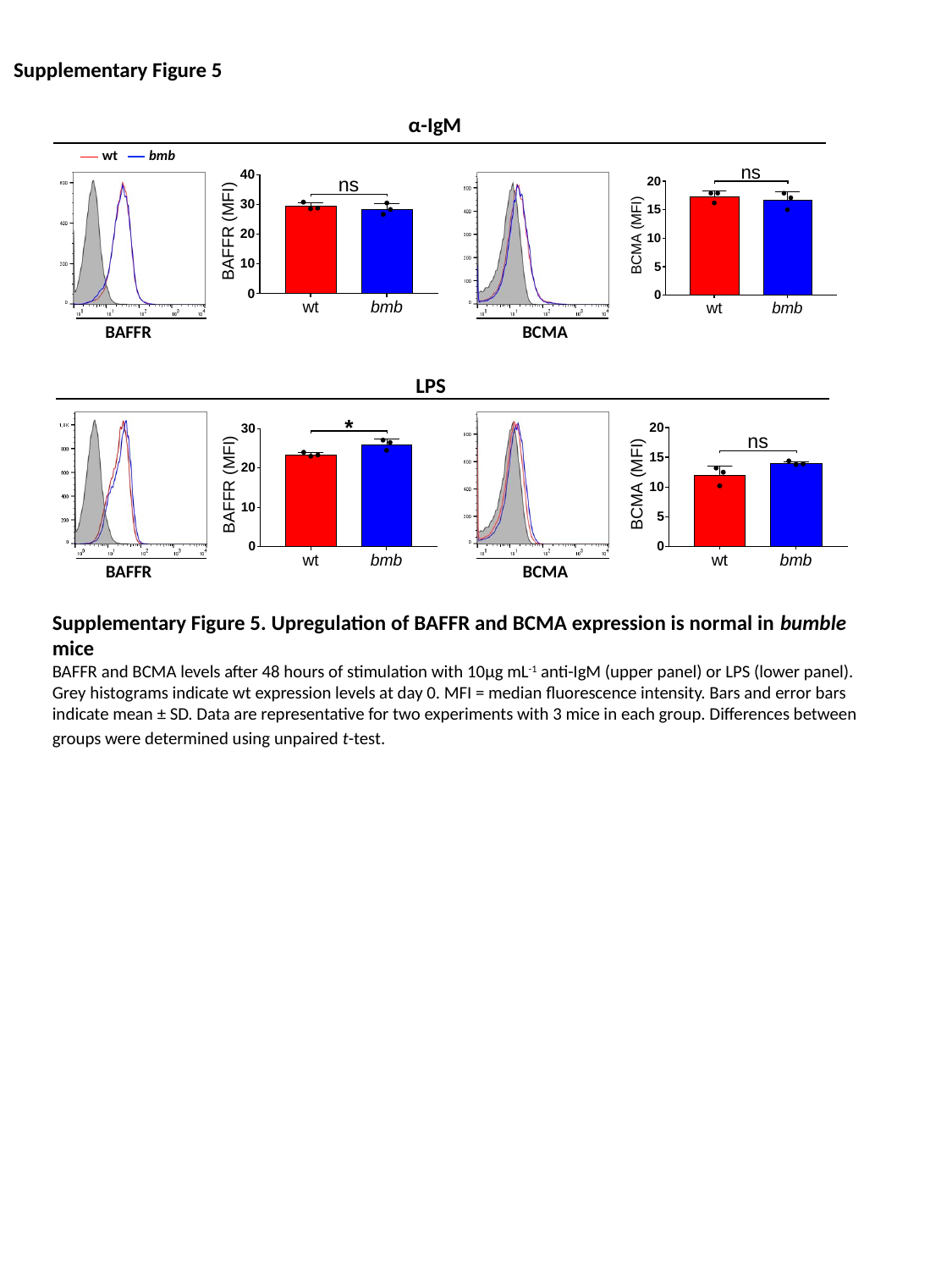

Supplementary Figure 5
α-IgM
wt
bmb
BAFFR
BCMA
LPS
BAFFR
BCMA
Supplementary Figure 5. Upregulation of BAFFR and BCMA expression is normal in bumble mice
BAFFR and BCMA levels after 48 hours of stimulation with 10μg mL-1 anti-IgM (upper panel) or LPS (lower panel). Grey histograms indicate wt expression levels at day 0. MFI = median fluorescence intensity. Bars and error bars indicate mean ± SD. Data are representative for two experiments with 3 mice in each group. Differences between groups were determined using unpaired t-test.

## Slide 6
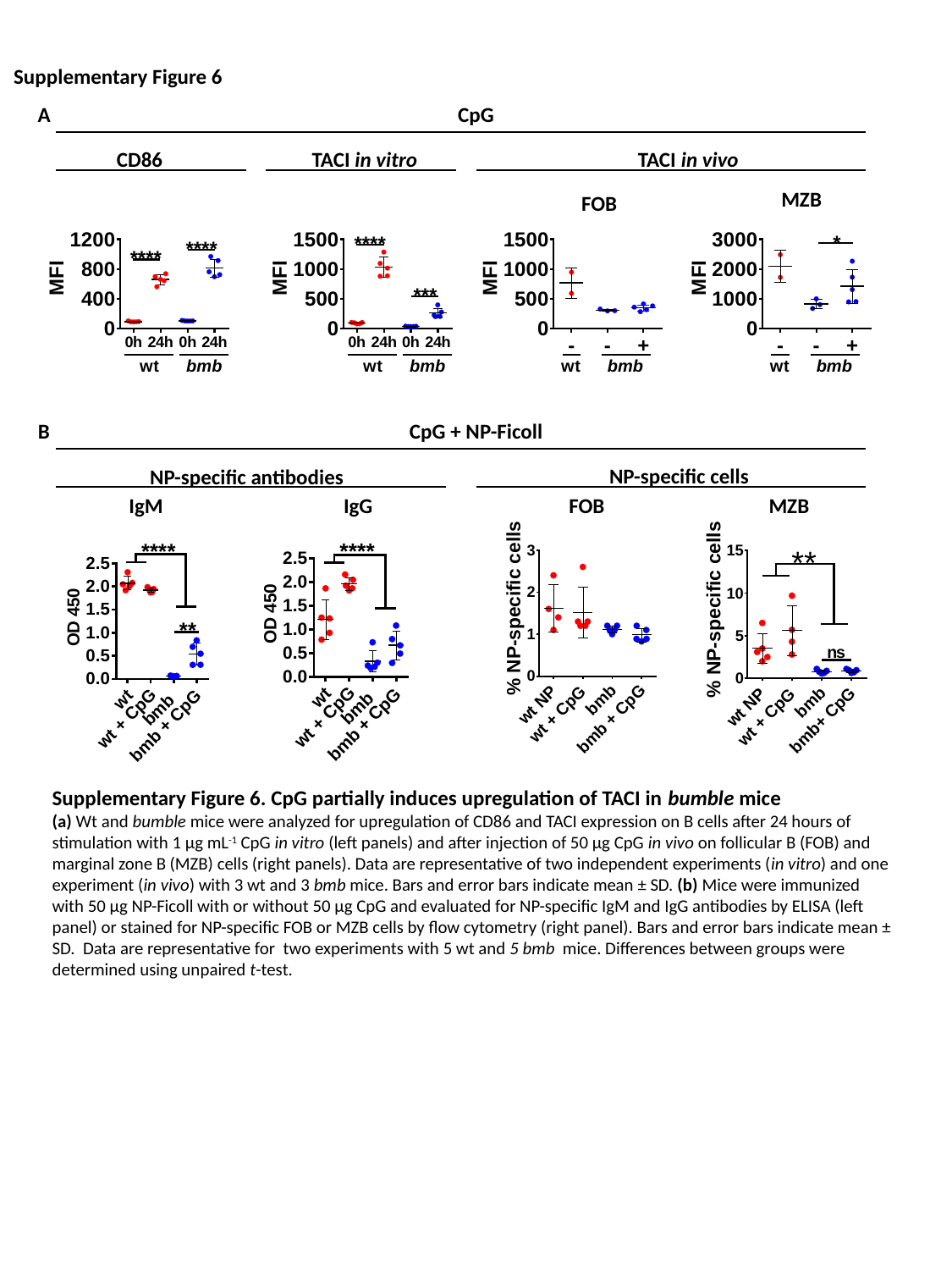

Supplementary Figure 6
A
CpG
CD86
TACI in vitro
TACI in vivo
MZB
FOB
CpG + NP-Ficoll
B
NP-specific cells
NP-specific antibodies
IgM
IgG
FOB
MZB
Supplementary Figure 6. CpG partially induces upregulation of TACI in bumble mice
(a) Wt and bumble mice were analyzed for upregulation of CD86 and TACI expression on B cells after 24 hours of stimulation with 1 μg mL-1 CpG in vitro (left panels) and after injection of 50 μg CpG in vivo on follicular B (FOB) and marginal zone B (MZB) cells (right panels). Data are representative of two independent experiments (in vitro) and one experiment (in vivo) with 3 wt and 3 bmb mice. Bars and error bars indicate mean ± SD. (b) Mice were immunized with 50 μg NP-Ficoll with or without 50 μg CpG and evaluated for NP-specific IgM and IgG antibodies by ELISA (left panel) or stained for NP-specific FOB or MZB cells by flow cytometry (right panel). Bars and error bars indicate mean ± SD. Data are representative for two experiments with 5 wt and 5 bmb mice. Differences between groups were determined using unpaired t-test.

## Slide 7
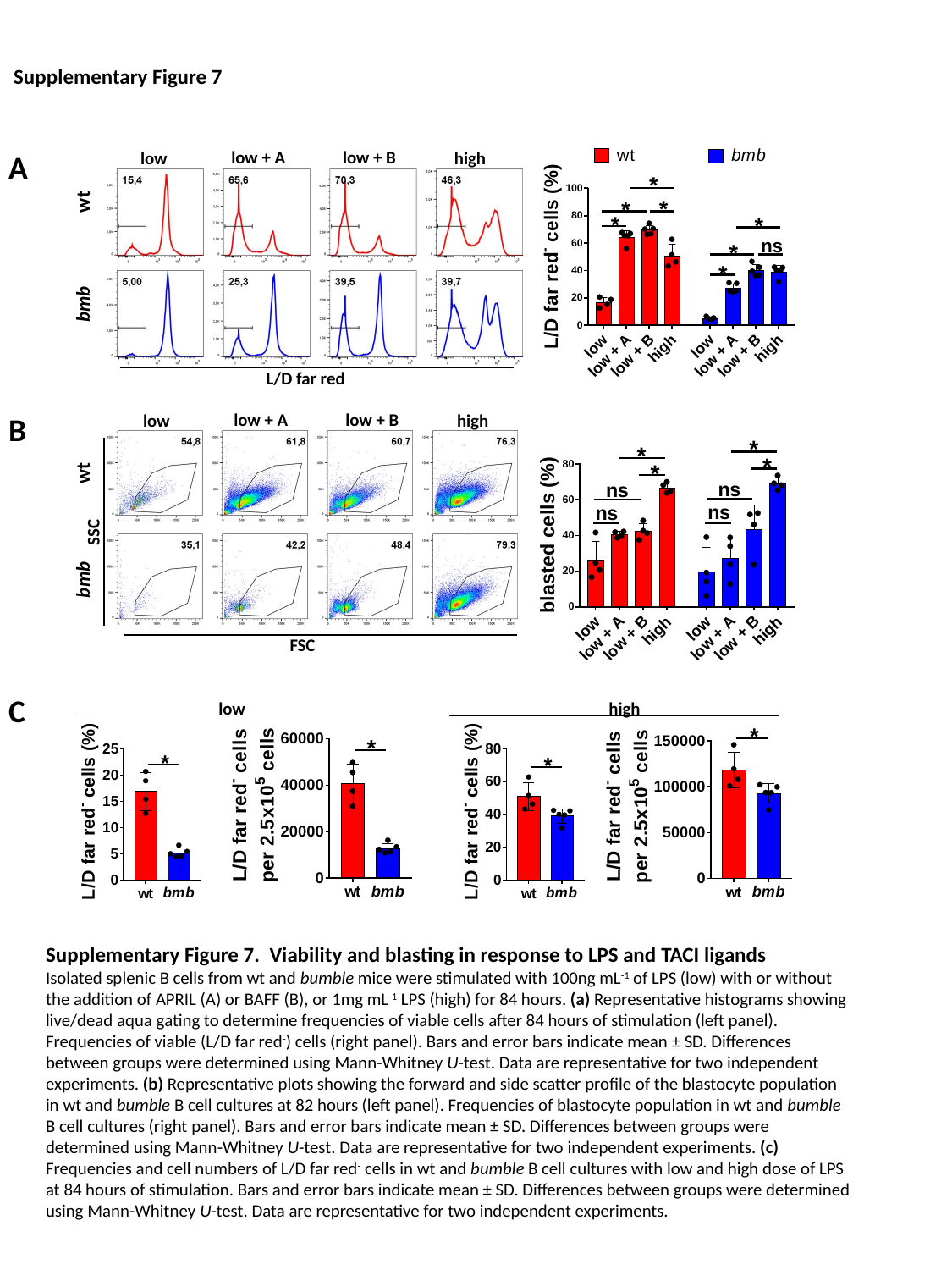

Supplementary Figure 7
low + A
low + B
A
low
high
wt
bmb
L/D far red
low + A
low + B
B
low
high
wt
SSC
bmb
FSC
C
low
high
Supplementary Figure 7. Viability and blasting in response to LPS and TACI ligands
Isolated splenic B cells from wt and bumble mice were stimulated with 100ng mL-1 of LPS (low) with or without the addition of APRIL (A) or BAFF (B), or 1mg mL-1 LPS (high) for 84 hours. (a) Representative histograms showing live/dead aqua gating to determine frequencies of viable cells after 84 hours of stimulation (left panel). Frequencies of viable (L/D far red-) cells (right panel). Bars and error bars indicate mean ± SD. Differences between groups were determined using Mann-Whitney U-test. Data are representative for two independent experiments. (b) Representative plots showing the forward and side scatter profile of the blastocyte population in wt and bumble B cell cultures at 82 hours (left panel). Frequencies of blastocyte population in wt and bumble B cell cultures (right panel). Bars and error bars indicate mean ± SD. Differences between groups were determined using Mann-Whitney U-test. Data are representative for two independent experiments. (c) Frequencies and cell numbers of L/D far red- cells in wt and bumble B cell cultures with low and high dose of LPS at 84 hours of stimulation. Bars and error bars indicate mean ± SD. Differences between groups were determined using Mann-Whitney U-test. Data are representative for two independent experiments.

## Slide 8
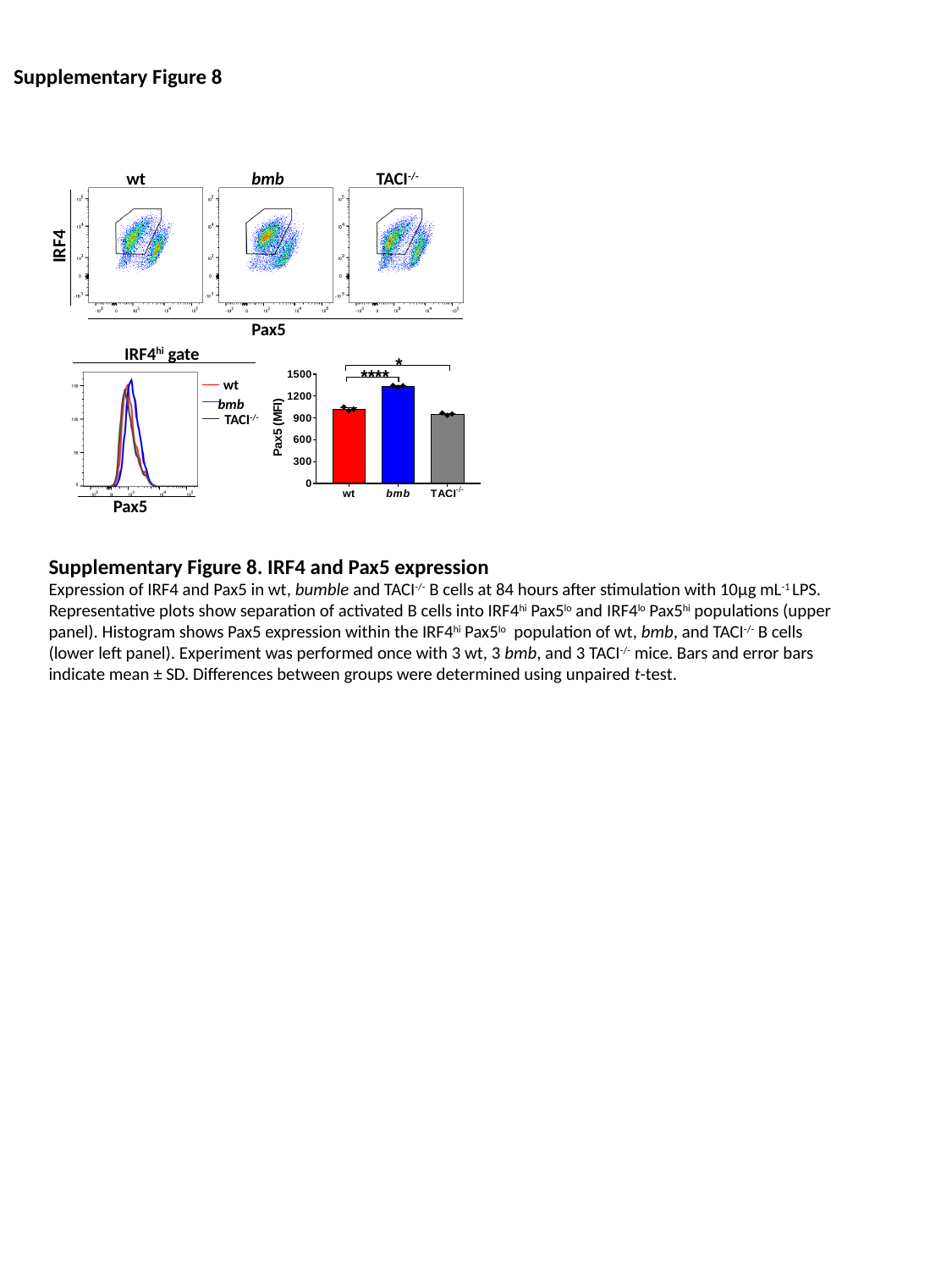

Supplementary Figure 8
wt
bmb
TACI-/-
IRF4
Pax5
IRF4hi gate
wt
bmb
TACI-/-
Pax5
Supplementary Figure 8. IRF4 and Pax5 expression
Expression of IRF4 and Pax5 in wt, bumble and TACI-/- B cells at 84 hours after stimulation with 10μg mL-1 LPS. Representative plots show separation of activated B cells into IRF4hi Pax5lo and IRF4lo Pax5hi populations (upper panel). Histogram shows Pax5 expression within the IRF4hi Pax5lo population of wt, bmb, and TACI-/- B cells (lower left panel). Experiment was performed once with 3 wt, 3 bmb, and 3 TACI-/- mice. Bars and error bars indicate mean ± SD. Differences between groups were determined using unpaired t-test.

## Slide 9
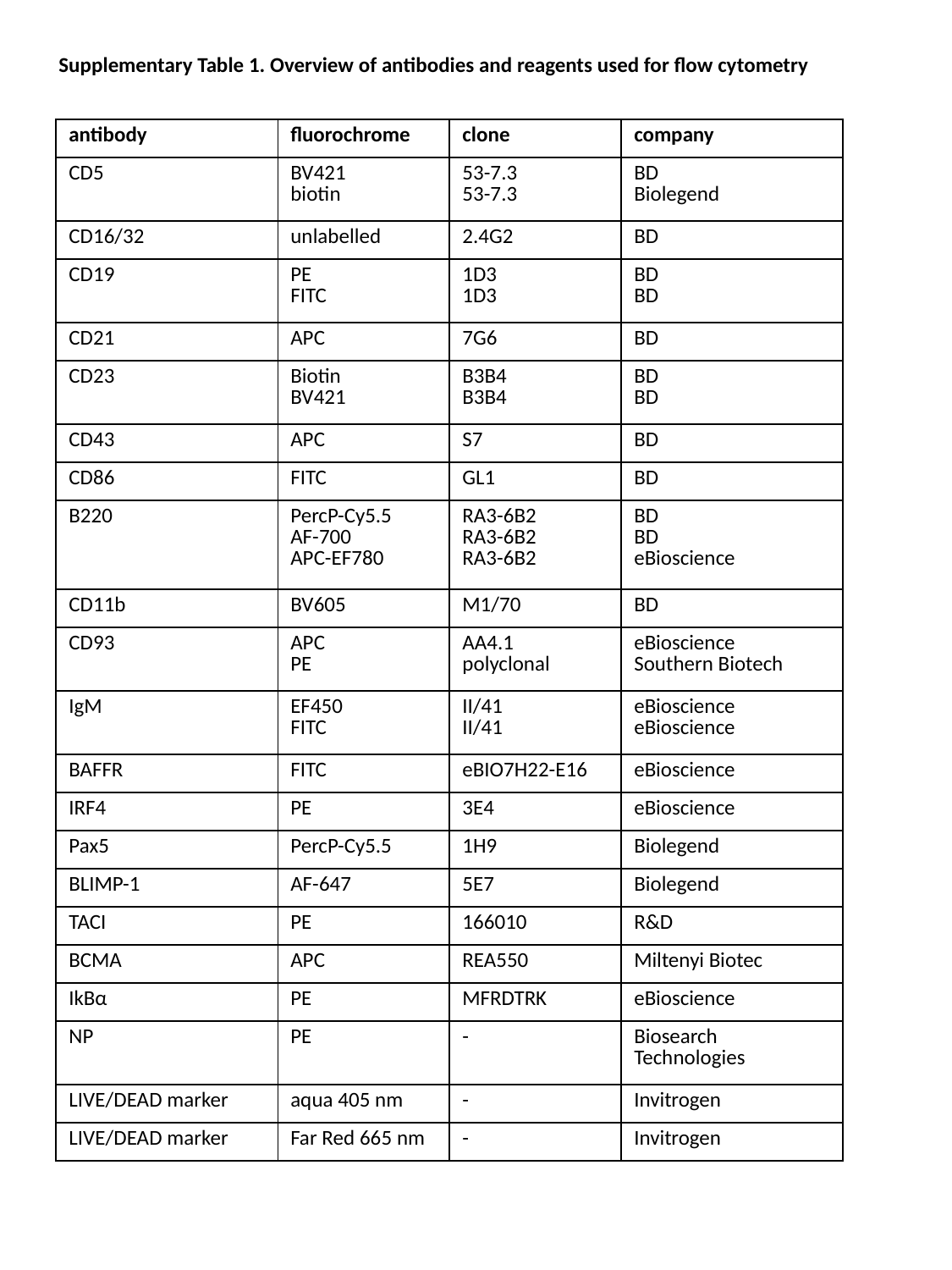

Supplementary Table 1. Overview of antibodies and reagents used for flow cytometry
| antibody | fluorochrome | clone | company |
| --- | --- | --- | --- |
| CD5 | BV421 biotin | 53-7.3 53-7.3 | BD Biolegend |
| CD16/32 | unlabelled | 2.4G2 | BD |
| CD19 | PE FITC | 1D3 1D3 | BD BD |
| CD21 | APC | 7G6 | BD |
| CD23 | Biotin BV421 | B3B4 B3B4 | BD BD |
| CD43 | APC | S7 | BD |
| CD86 | FITC | GL1 | BD |
| B220 | PercP-Cy5.5 AF-700 APC-EF780 | RA3-6B2 RA3-6B2 RA3-6B2 | BD BD eBioscience |
| CD11b | BV605 | M1/70 | BD |
| CD93 | APC PE | AA4.1 polyclonal | eBioscience Southern Biotech |
| IgM | EF450 FITC | II/41 II/41 | eBioscience eBioscience |
| BAFFR | FITC | eBIO7H22-E16 | eBioscience |
| IRF4 | PE | 3E4 | eBioscience |
| Pax5 | PercP-Cy5.5 | 1H9 | Biolegend |
| BLIMP-1 | AF-647 | 5E7 | Biolegend |
| TACI | PE | 166010 | R&D |
| BCMA | APC | REA550 | Miltenyi Biotec |
| IkBα | PE | MFRDTRK | eBioscience |
| NP | PE | - | Biosearch Technologies |
| LIVE/DEAD marker | aqua 405 nm | - | Invitrogen |
| LIVE/DEAD marker | Far Red 665 nm | - | Invitrogen |
